# IL-8 activates fibroblasts to promote the invasion of HNSCC cells via STAT3-MMP1

**DOI:** 10.1038/s41420-024-01833-7

**Published:** 2024-02-06

**Authors:** Yu Chen, Li Huang, Rui-Huan Gan, Shuo Yuan, Ting Lan, Dali Zheng, You-Guang Lu

**Affiliations:** 1 Department of Preventive Dentistry, Affiliated Stomatological Hospital, Fujian Medical University, 246 Yang Qiao Middle Road, Fuzhou, 350000 China; 2 Fujian Key laboratory of Oral Diseases, School and Hospital of Stomatology, Fujian Medical University, 88 Jiaotong Rd, Fuzhou, 350004 China; 3 Department of Oral and Maxillofacial Surgery, Affiliated First Hospital of Fujian Medical University, 20 Cha Zhong Road, Fuzhou, 350005 China

**Keywords:** Cancer microenvironment, Oral cancer

## Abstract

Matrix metalloproteinase-1 (MMP1) has an aberrant expression relevant to various behaviors of cancers. As dominant components of the tumor stroma, fibroblasts constitute an important source of Matrix metalloproteinase (MMPs) including mainly MMP1. The impacts of MMP1 derived from fibroblasts in tumor microenvironment, however, is not well defined. In this study, we demonstrated a part of crosstalk between fibroblasts and cancer cells that enhanced the invasiveness of cancer cells, IL8-induced activation of STAT3 signaling pathway as a key promoter to elevated MMP1 level in fibroblasts that supports the migration and invasion of head and neck squamous cell carcinoma (HNSCC) cells by extracellular matrix degradation. Importantly, once exposed to the inhibitor of STAT3 phosphorylation (TPCA-1), the enhanced induction of HNSCC cells invasion triggered by fibroblasts was significantly impaired.

## Introduction

Head and neck cancer is the sixth most common cancer worldwide [1]. Head and squamous cell carcinoma (HNSCC), the majority of these cancers, are usually diagnosed at an advanced stage with local-regional metastases, and their treatment remains a clinical challenge [2]. Recurrence and metastasis of malignant tumors remain the cause of adverse reactions to HNSCC therapy, which leads to adverse outcomes, including the occurrence of recurrence [3].

The tumor microenvironment (TME) has been an important factor in promoting disease progression and clinical recurrence in patients with HNSCC [2]. Fibroblast is a dominant cell type in TME, and for more than a decade, many studies have shown that these cells have a marked functional role in cancer metastasis [4]. Cancer-associated fibroblasts (CAFs) have been frequently mentioned over the past decade and are recognized as a key component of tumor progression. Evolving evidence suggests that they may contribute to extensive fibrotic matrix procedures in many different tumors [5]. In the TME, cancer cells begin to activate CAFs and increase their secretory activity, such as producing soluble factors. In a result, CAFs promote tumor cell proliferation, invasion, and spread to distant organs [6]. The lack of precise fibroblast-specific markers poses a challenge in studying the origin of CAFs. Therefore, when neither normal tissue-resident fibroblasts nor CAFs can be defined by markers, it is difficult to make hypotheses about the exact origin of CAFs [7]. Moreover, increasing distinct biomarkers for identifying CAFs have been reported recently. CAFs of various subsets would harbor pro-tumoral or anti-tumoral properties respectively, or both [8]. To partially avoid this restriction, our study tried to document the crosstalk between fibroblasts component and cancer cells in the TME of HNSCC.

CAFs enhance tissue invasion, metastasis, and proliferation through secretion of matrix components and metalloproteases (MMPs)-mediated ECM remodeling through cell surface mitogen shedding and mobilization of ECM-embedded growth factors [6]. Elevated levels of matrix metalloproteinases can be used as biomarkers to evaluate various types of cancer. Several clinical studies have demonstrated the promising aspects of natural matrix metalloproteinases inhibitors (MMPIs) expression but a large part of uncharted territory has been remained [9, 10]. Matrix metalloproteinase-1 (MMP1) has an aberrant expression relevant to various behaviors of cancer. MMP1 has been found with elevated expression in series primary cancers and its prognostic value has been revealed in several cancers [11]. Thus, fibroblasts constitute an main source of matrix metalloproteinases, especially including MMP1 [10]. The impacts of MMP1 derived from fibroblasts in TME is not well studied and documented.

STATs play an important role in tumor progression [12]. As a family member, STAT3 is canonically activated by tyrosine-phosphorylated downstream of numerous cytokines and accordingly has an continuous activation in a high proportion of tumors [13]. Other than its known roles in promoting tumor progression, it has recently been discovered that IL6-dependent STAT3 signaling plays a central role in inflammation-mediated cancer, obesity or metabolic reprogramming, cancer stem cells, and pre-metastatic niche formation [14]. Activated STAT3 acts as a transcriptional co-activator of RelB to positively regulate MMP1 in colon cancer [15] and induced M2 macrophage polarization [16]. In PDAC, CAFs derived STAT3 promotes tumorigenesis [17]. IL-6 or IL-8 secreted by tumor cells induces the activation of STAT3 in NK cells and inhibits the function of NK cells in ESCC [18]. Although a series of studies have shown that STAT3 is an ideal target for cancer therapy, at present, effective therapeutic interventions to inhibit STAT3 and produce powerful anti-tumor effects in the clinical environment need to be further explored and developed [14]. In this study, we demonstrated that STAT3 signaling in fibroblasts is activated by HNSCC cell lines and identify a IL8/STAT3-driven markers that enrich the gene encoding the MMP1 protein. The inhibition to STAT3 significantly impaired fibroblast-induced tumor invasion in vitro.

## Results

### MMP1 is upregulated in HNSCC

**Fig. 1:**
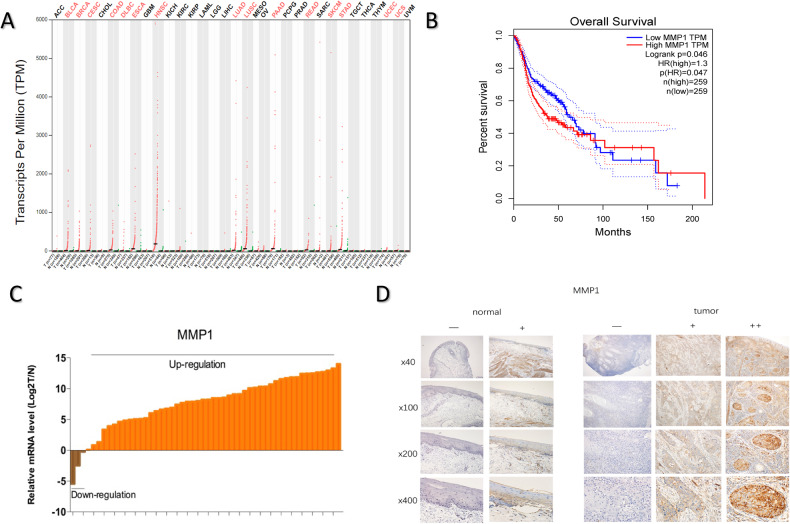
MMP1 is upregulated in HNSCC. **A** MMP1 expression profile across all tumor samples and paired normal tissues in TCGA. Compare with other primary tumor samples, MMP1 expression level was significantly upregulated in HNSCC samples. **B** Kaplan–Meier analysis of overall survival. Compared with patients with low MMP1 expression, those with high MMP1 expression had a significantly lower overall survival rate. **C**, **D** MMP1 expression in HNSCC samples and paired normal tissues (tumor/normal) from 53 patients were examined by qRT-PCR (brown for MMP1 down-regulation in tumor tissues compared to normal tissues while orange for up-regulation in tumor tissues) and those from another 92 patients were examined by immunohistochemistry staining. (−: negative; +: positive; ++: strongly positive).

Differential gene expression analysis in HNSCC dataset revealed MMP1 was on the over-expressed list of top 10 (Supplementary Table S1). An analysis of The Cancer Genome Atlas (TCGA) database revealed that MMP1 was esophageal carcinoma, pancreatic adenocarcinoma, lung squamous cell carcinoma and significantly upregulated especially in HNSCC samples across all tumor samples and paired normal tissues (Fig. 1A). We found high express of MMP1 were associated with poor overall survival (Fig. 1B). Quantitative real-time PCR (qPCR) of mRNAs for 53 tissues paired with normal parts (Fig. 1C) and immunohistochemical staining of 92 specimens containing paired non-tumor specimens (Fig. 1D) from HNSCC patients validated this finding.

### MMP1 enhances cancer cell migration

**Fig. 2:**
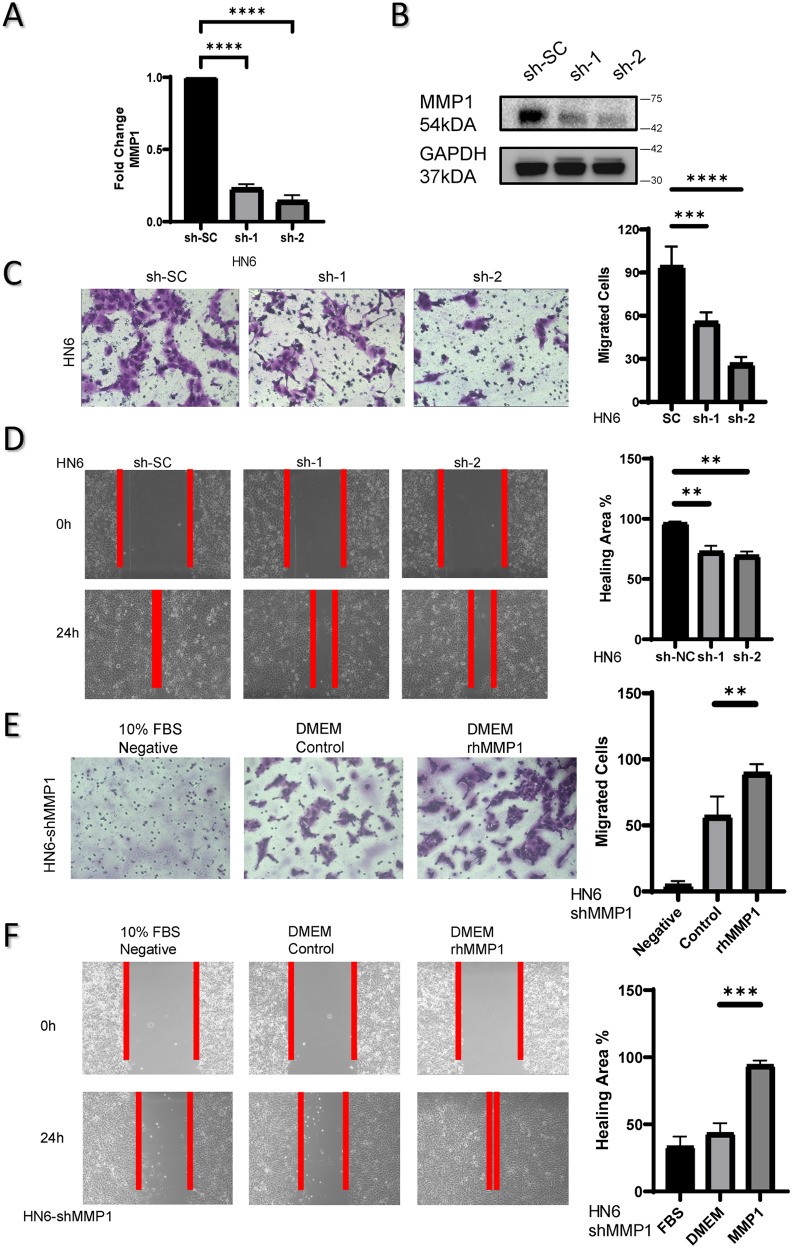
MMP1 enhances cancer cell migration. **A**, **B** The transcriptional and translational levels of MMP1 were detected by qRT-PCR and western blotting in HNSCC cell line (HN6). MMP1 was downregulated in both. The migration abilities of HNSCC cells with MMP1 knockdown were detected using Transwell assays (**C**) and wound-healing assays (**D**) respectively. The migration abilities of MMP-1-knockdown HNSCC cells treated with rhMMP1 (at 24 h, 20 ng/ml) were detected using Transwell assays (**E**) and wound-healing assays (**F**) respectively; Data are presented as the mean ± SD from three independent experiments (*p < 0.05, **p < 0.01, ***p < 0.001).

To assess the biological role of MMP1 in tumor cells, shRNA targeting the MMP1 gene acts on CAL27 and HN6 cell lines, detecting silencing by qRT-PCR and western blotting. Both of transcriptional level and post-translational level of MMP1 were downregulated in HNSCC cells (Fig. 2A, B). The migration abilities of CAL27 and HN6 cells with MMP1 downregulated were inhibited compared to the control group, as evaluated by the wound-healing and transwell assays. (Fig. 2C, D and Supplementary Fig. S1B, C). Further, rhMMP1 (20 ng/ml) was used to stimulate MMP1-downregulate-CAL27 and HN6 (Fig. 2E, F and Supplementary Fig. S1D, E), the results indicated that rhMMP1 promote the migration abilities of cells compared to control group. While there were no significant alterations in proliferation (Supplementary Fig. S1F).

### NFs-secreted MMP1 promotes the invasion of cancer cells

**Fig. 3:**
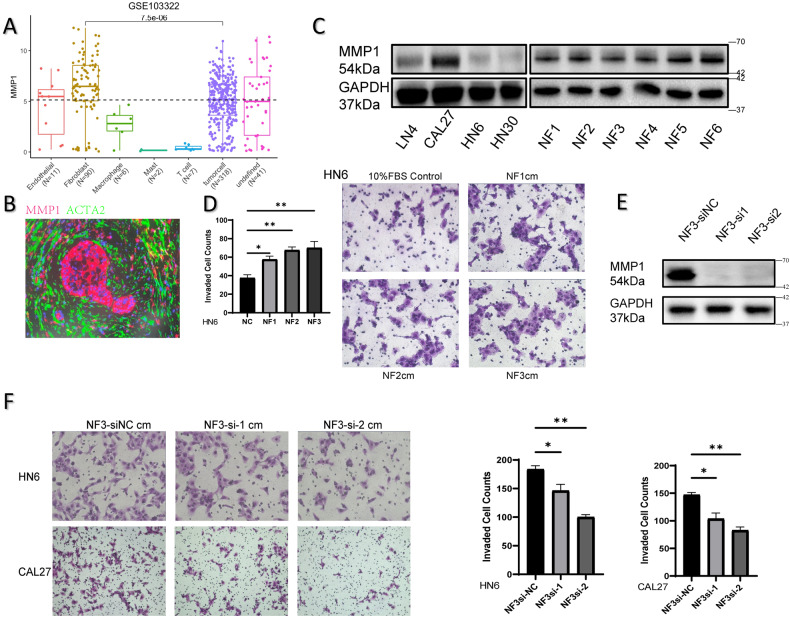
NFs-secreted MMP1 promotes the invasion of cancer cells. **A** MMP1 expression among identified cell types in single-cell RNA-seq dataset (GSE103322). **B** The immuno-colocalization of MMP1 (red) and ACTA2 (green) was detected in HNSCC samples by fluorescence microscope, and was represented as the overlapping fluorescence for each channel, where the combined pixels were yellow. **C** The expression of MMP1 protein levels in different HNSCC cell lines (LN4, Cal27, HN6, HN30) versus NFs. **D** The images of invaded transwell assay for HN6 cells (24 h) showed that each group of NF conditioned mediums promoted the invasive abilities of cancer cells compare to blank control (DMEM with 10% FBS). Each group of fibroblast was starved overnight and conditioned mediums were collected. **E** The downregulation of MMP1 protein expression in NF3 was demonstrated by western blotting. **F** Indirect co-culture invaded transwell assays were performed. Each group of NF3 fibroblasts were transfected with siRNA and cultured on the 24-well plates overnight, and the conditioned medium of each was collected for the lower layer of the matrigel-invasion chambers. The results showed fewer invasive tumor cells were detected compared to the siNC group. The chemotactic effect of NF3 on HN6 (24 h) and CAL27 (48 h) were attenuated with downregulation of MMP1.

It is widely proved that cancer progression and metastasis are closely related to the TME and malignant tumors may not depend solely on cancer cell-autonomous defects. We integrated the single cell RNA-seq data analysis from 18 patients with oral cavity tumors from Gene Expression Omnibus GSE103322, and found MMP1 highly expressed in both fibroblasts and tumor cells compare to other cell types (Fig. 3A). Immunofluorescent staining for MMP1 and myofibroblasts marker α-smooth muscle actin (ACTA2) revealed that the immuno-colocalization of MMP1 (red) and ACTA2 (green) was detected in stroma and MMP1 protein was highly expressed around the tumor cells (Fig. 3B). We further measured the expression of MMP1 in cancer cell lines and primary NFs from HNSCC patients. The results indicated that MMP1 was relatively highly expressed in fibroblasts (Fig. 3C). Then we found NFs enhanced the invasion ability of cancer cells in indirect co-culture condition (Fig. 3D). To validate whether fibroblast-derived MMP1 promotes the progress of cancer, the transwell assays were performed. As expected, MMP1-downregulate fibroblasts lost the function for promoting tumor cell invasion compared to SiNC group (Fig. 3F).

### Cancer cells upregulate the expression of MMP1 in NFs through activating STAT3

**Fig. 4:**
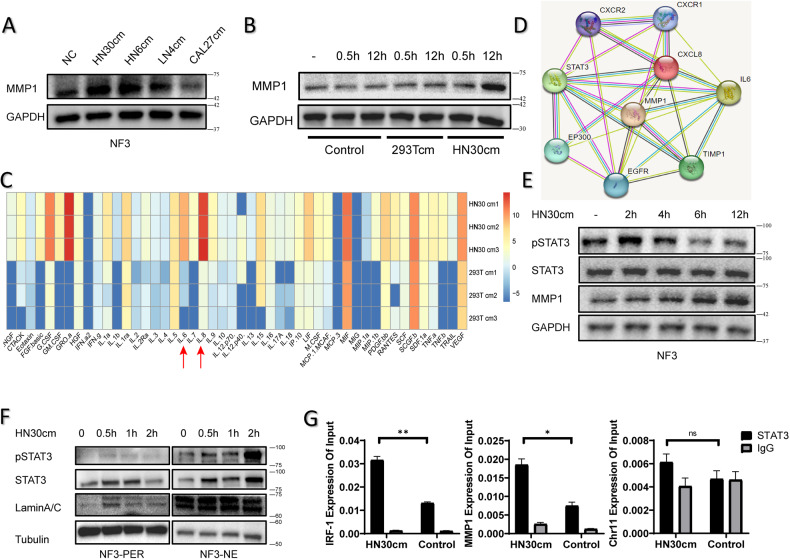
Cancer cells upregulate the expression of MMP1 in NFs through activating STAT3. **A**, **B** MMP1 proteins in NFs were detected at indicated time after CM treatment by western blotting assays. The expression of MMP1 protein levels in NFs was upregulated after 12-h HN30-CM treatment. **C** Heatmap of differential expression of selected proteins in culture supernatants (HN30-CM compared with 293T-CM). IL6 and IL8 were the higher components. **D** IL6, IL8 (CXCL8), STAT3 and MMP1 are relevant according to STRING network clusters. **E** HN30-CM induces STAT3 phosphorylation in NFs within 2 h, and upregulates the expression of MMP1 after 12 h. **F** HN30 conditioned medium increased phosphorylated STAT3 protein levels in nuclear fractions. **G** ChIP assay was used to assess the binding of STAT3 to the promoters of the indicated genes in NF3 or HN30-CM mediated (1 h for treatment) NF3 with anti-STAT3 antibodies, followed by qPCR analysis.

Previous investigations have confirmed that there were complex heterotypic interactions of cancer cells with other cell in tumor microenvironment (TME). Our studies found that CM from HN30 cell lines upregulate the expression of MMP1 in NFs (Fig. 4A). To determine the component of CM from HN30 upregulated the expression of MMP1 in NFs, CM from 293 T cell line was used as a negetive control. Western blotting for MMP1 proteins showed that, compared with HN30-CM, 293T-CM failed to increase the expression of MMP1 in indicated time (Fig. 4B, Supplementary Fig. S2A). After HN30 and 293 T were serum-starved for 48 h, the supernatants were collected for protein array. Heatmap of differential expression of selected proteins in culture supernatants showed that IL6 and IL8 were the higher components in HN30-CM compared with 293T-CM (Fig. 4C). The concentration of IL-6 and IL-8 in several cells conditioned mediums were also detected by ELISA assays (Supplementary Fig. S3A). Subsequently, a protein-protein interaction network was constructed for IL6, IL8 (CXCL8) and MMP1 through STRING, which revealed STAT3 was relevant in network clusters (Fig. 4D). HN30 conditioned medium induces STAT3 phosphorylation (Tyr705) in NFs within 2 h, and upregulates the expression of MMP1 after 12 h (Fig. 4E). Then, we went back and retested the sample in Fig. 4B and found compared with HN30-CM, 293T-CM failed to increase the expression of phospho-STAT3 in indicated time (Supplementary Fig. S2A). The analysis of subcellular fraction results showed that HN30-CM significantly increased phospho-STAT3 protein levels in nuclear fractions in 2 h (Fig. 4F). Immunofluorescent assays were performed to detected increased expression of phospho-STAT3 protein located in nuclear by confocal laser scanning microscope (Supplementary Fig. S2B). To confirm that MMP1 is a direct transcriptional target for STAT3, ChIP assays were used to detect that STAT3 binds to the MMP1 promoter region (Fig. 4G), compared with IRF1 (positive control) and Chr11 (negative control). Taken together, these findings suggest that CM from HN30 upregulated the expression of MMP1 in NFs through activating STAT3.

### IL8 activates STAT3 to promote secreted MMP1 in fibroblasts

**Fig. 5:**
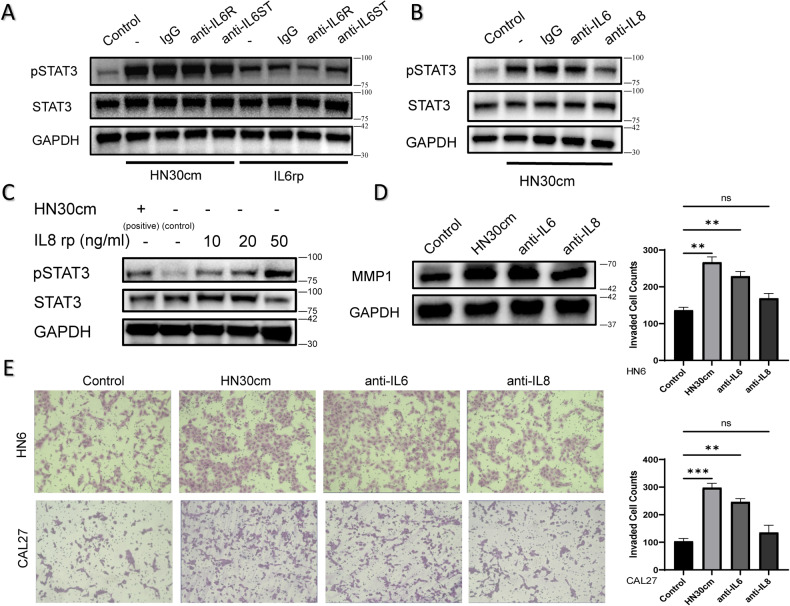
IL8 activates STAT3 to promote secreted MMP1. **A**–**C** Phospho-STAT3, STAT3 proteins were detected 0.5 h after treatment by western blotting assays. After NF3 cells were cultured in basal medium and starved overnight, IgG, anti-IL6R, anti-IL6ST or anti-IL6 and anti-IL8 (100 ng/ml) were added before treating with HN30-CM and IL6 recombinant protein (10 ng/ml) or IL8 recombinant protein (10-50 ng/ml) for 0.5 hours. **D** MMP1 proteins were detected 12 hours after HN30-CM or together with IL6 or IL8 antibody (100 ng/ml) treatment in NF3 cells by western blotting assays. **E** The images of indirect co-culture invaded transwell assays. Invasion of HN6 and CAL27 cells cultured with NF3 (control without treatment), NF3 pretreated with HN30-CM (positive), NF3 pretreated together with IL6 antibody and HN30-CM, NF3 pretreated together with IL8 antibody and HN30-CM.

As indicated in our previous findings, both IL6 (3.5 ng/ml) and IL8 (11.2 ng/ml) are highly secreted in HN30-CM. Next, we tried to determine which of these two components was responsible. Anti-IL6R, anti-IL6ST or anti-IL6 and anti-IL8 were used as comparative combination of IL6 or IL8 protein in HN30-CM. The results showed anti-IL8 suppressed the function of HN30-CM (Fig. 5A, B). Compared to positive and control group, IL8 recombinant protein did cause activation of STAT3 phosphorylation in NF3 (Fig. 5C) and blocking IL8 suppressed MMP1 expression in NF3 induced by HN30-CM (Fig. 5D). The chemotactic ability of NF3 to HN6 and CAL27 was enhanced by HN30-CM and weakened by blocking IL8 (Fig. 5E).

### The inhibitor of STAT3 signaling pathway impairs NFs ability to support invasion of cancer cells

**Fig. 6:**
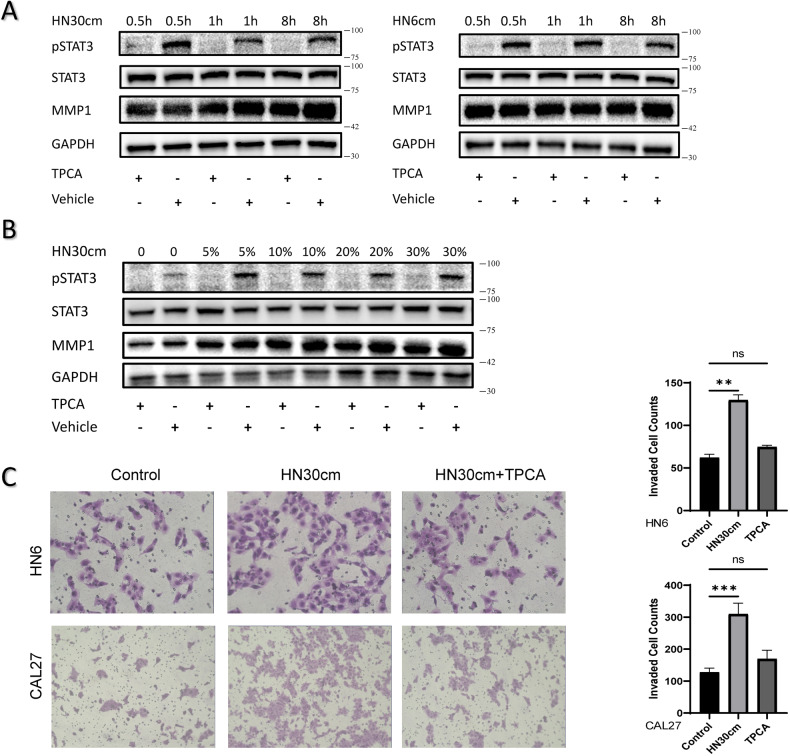
The inhibitor of STAT3 signaling pathway impairs NFs ability to support invasion of cancer cells. **A**, **B** Phospho-STAT3, STAT3 and MMP1 proteins are detected in indicated time by western blotting assay. NF3 cells were serum-starved and **A** treated with CM from HN30 and TPCA (inhibitor of STAT3 signaling pathway) for the indicated time or **B** treated with TPCA and CM from HN30 with indicated contents for 12 hours. **C** Invasion of HN6 cells cultured with NF3, NF3 pretreated with CM from HN30 cells or NF3 pretreated with TPCA and CM from HN30 cells.

TPCA-1 is an potent inhibitor of STAT3 phosphorylation. In the present study, without changing total STAT3 levels, 0.5 μM TPCA-1 completely inhibited NFs STAT3 phosphorylation induced by HN30-CM in time-dependent manner (Fig. 6A). Similarly, HN30-CM increased MMP1 expression in dose-dependent manner after 12 hours, which was then suppressed by TPCA-1 (Fig. 6B). The results with a similar tendency were obtained for HN6-CM (Fig. S2C). Once exposed to TPCA, the enhanced induction of HNSCC cells invasion triggered by NFs was significantly impaired (Fig. 6C).

### WPOI progression and lymph node metastasis are associated with the MMP1 derived from fibroblasts

**Fig. 7:**
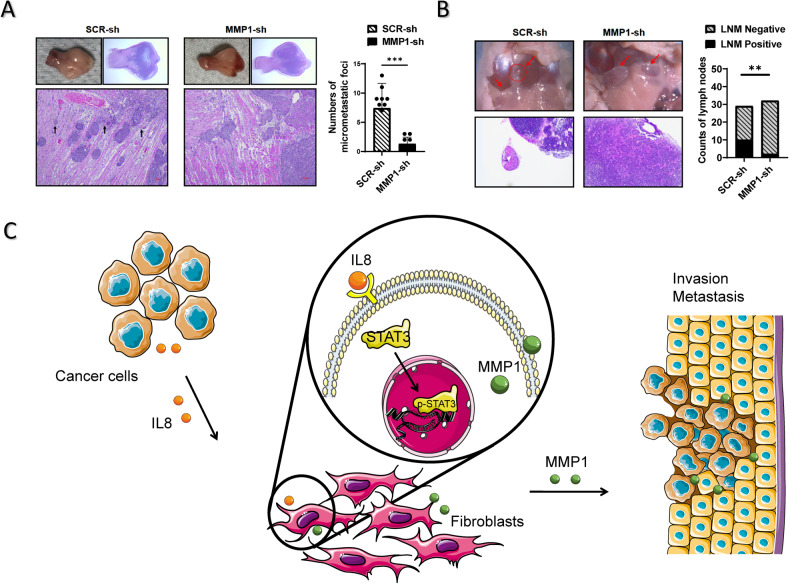
WPOI progression and lymph node metastasis are associated with the MMP1 derived from fibroblasts. **A**, **B** Representative H&E images and graphical analysis of frequency of micrometastatic foci and lymph node metastasis. After 106 LN4 OSCC cells were mixed with 106 NF-SCR-sh or NF-MMP1-sh and injected into the tongue of 5-week-old nude mice. n = 10/group. **, P < 0.01; ***, P < 0.005; P = two tailed t test or Fisher’s exact test. **C** Proposed working model for the prometastatic roles of MMP1 and STAT3 in fibroblasts. Cytokine IL-8 is secreted by tumor cells, which activates fibroblasts around. Thus, in response to IL-8, STAT3 in fibroblasts are phosphorylated and translocated into the nucleus. As a result, the STAT3-dependent MMP1 gene encoding secreted proteins is induced. Those in turn exert pro-migratory and pro-invasive functions on tumor cells.

To investigate the function of fibroblast-derived MMP1 in tumor progression, we established a mouse model of orthotopic transplantation of oral squamous cell carcinoma. We co-injected human oral squamous cell carcinoma cell lines and fibroblasts LN4/NF-SCR-sh and LN4/NF-MMP1-sh into the tongue. The results revealed that fibroblasts with knockdown of MMP1 significantly decreased the incidence of micro-metastases foci (cellular dissociation in small cellular groups, n < 15) and lymph node metastasis in the tongue transplant (Fig. 7A, B), suggesting that MMP1 originating from fibroblasts is implicated in the invasive and metastatic phenotype of tumors.

## Discussion

As a member of the peptidase M10 family of matrix metalloproteinases, MMP1 was proved to play key roles in a variety of cancers by remodeling the extracellular matrix. MMP1 is responsible for the digestion of types I, II, and III of natural fibrous collagen in the extracellular environment [19]. MMP1 antibody-conjugated material can be used to diagnose cancers with high concentrations of MMP1 [20]. Althrough elevated expression of MMP1 was observed in both cancer and stromal cells, it is not clear which type of cells it mainly comes from. In the present study, we found MMP1 highly expressed in fibroblasts compared with HNSCC cell lines; and MMP1 from cancer cells had little effect to promote the tumor progression except for the migration ability, whereas MMP1 from fibroblasts enhanced the invasiveness of cancer cells.

Furthermore, we demonstrated that the secreted MMP1 from fibroblasts was increased by those cancer cells without a high concentration of MMP1. Recent studies continue to indicate that, in TME, activated fibroblasts play multiple roles in cancer progression with active secretome, including secreting growth factors or other cytokine into ECM [4]. Due to the heterogeneity and the constant crosstalk between the TME and the tumor cells, one may speculate, just like T cell differentiation, in order to possess diverse activities, some quiescent fibroblasts may be able to differentiate into different subsets of functional fibroblasts [4]. A variety of inflammatory modulators can activate CAFs, among which interleukin-1 mainly comes into effect through NF-κB and IL-6 mainly activates the STATs family [21]. Crosstalk between tumor and CAFs together with positive feedback involving JAK/STAT signaling, changes in contractable cytoskeleton, and histone acetylation promote the further activation of CAFs [7]. As the proinvasive properties of CAF remain activated even after their isolation from tumors and propagation [21]. On the other hand, according to several studies, α-SMA+ or S100A4+ CAFs are also potentially anti-tumor [8]. So, it comes to be widely accepted that CAFs have a “double face” [8]. Unlike CAFs, normal fibroblasts are generally activated for physiological repair or fibrosis [4], which is considered as an anti-tumoral effect preciously. However, activated fibroblasts would produce proteases that are involved in ECM remodeling [4], which could be associated with enhanced tumor motility. Instead of CAFs, normal fibroblasts (NFs) turn out to be our starting point. Here, we show that those NFs, isolated from HNSCC patients, results from the cancer cells-mediated activation of STAT3, which regulates MMP1 expression that are subsequently utilized by the cancer cells.

Constitutive activity of STAT3 is frequently observed in cancer cells. During these years, several unexpected fresh members and potential mechanisms of the JAK-STAT3 pathway have emerged in types of cancer [22]. In addition, enhanced STAT3 activity plays a central role in tumor progression, both through the way tumor cells are intrinsic and through its ability to regulate the environmental activity of surrounding cells [23]. STAT3 supports the protumor activity of CAFs, which induces the production and release of soluble factors into CM [6]. The strong responsiveness of MMP1 to EGF relies on STAT3 phosphorylation and its interaction with c-JUN, and with this crucial activation of STAT3, T24 cells exhibit malignant characteristics in bladder cancer. In addition, the phosphorylated form of STAT3 can only be combined with the stat element of the MMP1 promoter when it is combined with the c-JUN and AP-1 elements [24]. Our ChIP experiments and western bloting for nuclear and cytoplasmic extractions clearly detected STAT3 binding to MMP1 promoter regions when phosphorylated form of STAT3 translocated into the nucleus.

To understand how cancer cells activates NFs, we analyzed the components of the CM from cancer cells and found IL6 and IL8 were highly secreted. The GP130 receptor β subunit is critical in serving as a typical signal for STAT3 in response to IL-6 family, and activation of the complex can induce the expression of enzymes required by cells for cell migration and exomatrix remodeling, which can promote wound healing and tumor progression [25]. While IL6/IL8-JAK2 signaling was reported to induce BRD4 activation in colorectal cancer, leading to chromatin remodeling and resistance to BETi treatment. IL6/IL8 secreted CAFs and associated JAK2-STAT3 signaling result in cancer relapse and poor prognosis [26]. In our case, it is anti-IL8 rather than anti-IL6 that downregulated the phosphorylated STAT3 in fibroblasts, and as a result, MMP1 was not upregulated.

Combined with the above results and those supported by other studies [27, 28], we come to conclude that STAT3 blockade could be a promising therapeutic strategy for HNSCC. Phosphorylation and transactivation of STAT3 induced by cytokines and non-receptor tyrosine kinase are disrupted by TPCA-1 in dose- and time-dependent manner [29]. When elevated levels of phosphorylated STAT3 in NFs were eliminated upon TPCA-1 treatment, in our case, MMP1 was not upregulated and the invasiveness of cancer cells was not promoted.

In summary, we demonstrated a part of crosstalk between NFs and cancer cells that enhanced the invasiveness of cancer cells. In this context, IL8-induced activation of STAT3 signaling pathway as a key promoter to elevated MMP1 level in fibroblasts, supports the migration and invasion of HNSCC cells by ECM degradation (Fig. 7C), which suggests that targeting IL8-STAT3-MMP1 axis could be considered to resist the metastasis of HNSCC.

## Material and methods

### Clinical samples and cell culture

92 paraffin-embedded tongue cancer specimens and 53 paired fresh HNSCC specimens detected MMP1 expression. Normal fibroblasts (NFs) and cancer-associated fibroblasts (CAFs) were derived from another 7 pairs of fresh tissue samples from patients in the First Affiliated Hospital of Fujian Medical University. Our study was approved by the Institutional Review Committee of Fujian Medical University with the written informed consent of each participant. CAL27, HN6 and HN30 cell lines were purchased from ATCC (American Type Culture Collection). LN4 is a highly metastatic type of CAL27 [30]. Cells are cultured in the recommended medium and cultured at 37 °C and 5% CO_2_.

### RNA extraction and quantitative real-time PCR analysis

Use TRIzol reagent (#15596018; Invitrogen, Carlsbad, USA), and quantified the purity and concentration of RNA with ultraviolet spectrophotometry. After the measurement, the RNA was diluted separately into RNASE-free water and then used using the PrimeScript RT Kit (#RR037A; Beans, Japan). Use SYBR Premix Ex Taq (#RR420A; Takara) according to the manufacturer’s instructions. The expression of each gene is normalized to the GAPDH mRNA level of each sample obtained from parallel experiments. The primers for amplification are listed in Supplementary Table S3. Data analysis was performed using the 2-ΔΔCt method.

### Immunohistochemical staining assay

The immunohistochemical approach was performed and MMP1 staining was quantified as previously described [30]. Primary antibody against MMP1 (1:2000; ab137332; Abcam) was used.

### RNA interfere and plasmid transfection

12 h prior to transfection, exponential phase cell digestion counts are loaded into 6-well plates at a ratio of 3 ×10^5^ or 1 ×10^5^ cells/well, respectively. Then transfect cells with plasmid (pCDH-CMV-MCS-EF1-Puro-MMP1, TongYong, Anhui, China) using Lipofectamine 3000 (1713234, Invitrogen) or Lipofectamine RNAiMAX (1044526, Invitrogen).

### Lentiviral transduction

Negative controls (shRNA-SC) and MMP1 (sh1-3) lentiviral vector constructors were produced by Shanghai Genechem Co., Ltd., China. Perform lentiviral transduction of 2 cell lines according to the instructions. After 48 h, effectively infected cells were screened using medium with a final concentration of 2 ug/mL of puromycin. Stably stained cells were screened after being passaged two to three times.

### Nuclear/cytoplasmic extraction and western blotting assay

Cells were harvested with trypsin-EDTA and then centrifuged and resuspended in cold buffer A (10 mM HEPES pH 7.9, 10 mM KCl 0.1 mM EDTA, 0.1 mM EGTA 1 mM DTT, 1x protease inhibitor cocktail). Then after swelling on ice for 15 min, 10% solution of NP-40 was added and the tube was vigorously vortexed for 10 s. The cells lysate was centrifuged at 12,000 g for 1 min, and the supernatant was transferred as cytoplasmic extract. The nuclear pellet was resuspended in RIPA buffer. The conventional cytosolic protein was separated and transferred to PVDF membranes, subsequently examined by immunoblotting as previously described [30]. Primary antibodies against MMP1 (#54376; CST), Phospho-STAT3 Tyr705 (#9145; CST), STAT3 (#12640; CST), GAPDH (HRP-60004; Proteintech, China), Lamin A/C (10298-1-AP; Proteintech, China), Alpha Tubulin (HRP-66031; Proteintech, China) in 3% bovine serum albumin and secondary antibody (Mouse BA1050; Rabbit BA1054; Boster, China) were used. Protein bands were visualized using super enhanced chemiluminescence prime (S6008L; Bioscience, China).

### Cell viability assay

Cells were transfected with siRNAs for 24 h and seeded in each plate at a density of 2000 cells per well. The viability of the cells was analyzed using the Cell Counting Kit-8 (Dojindo Molecular Technologies, Japan).

### Wound-healing assay

We seeded cells into 12-well plates and incubated them until they became confluent. A P200 tip was used to scrape the cells. Then cells were washed with basal medium for 3 time and incubated with serum-free basal medium for more 48 h. At 24–48 h, nonoverlapping field images were collected.

### Transwell migration and invasion assays

Invasion and migration assays were performed in transwell inserts with polypropylene terephthalate membranes (24-well inserts, 8 mm in diameter, Corning). Migration transwells were filled with a suspension containing from 5 ×10^4^ cells per 500 μl. For chemo-attraction, 700 μl of medium containing 10% FBS or CM (cells’ conditioned medium), would be placed into the bottom of the well. The cells on the lower surface of the insert were stained with 0.5% crystal violet 24–48 h later. A similar procedure was performed as described above, except that matrigel was pre-coated onto the upper chamber for transwell invasion assays (24-well plate, 354480, Corning). A microscope camera (Axio Observer, ZEISS) was used to visualize and photograph staining cells. A random sample of five fields was selected, and the cells counted three times. The data are presented as means with standard deviations.

### ELISA assays and conditioned medium treatment

Cells were seeded in 6-well plates overnight, with or without other intervention. Then the medium was replaced by FBS-free DMEM which would be collected as a conditioned medium after 24 h. The concentration of IL-6/IL-8 in a conditioned medium was detected by ELISA kits (LCSED10377 and ED-10380; LunChangShuo Biotech; China) according to the manufacturer’s instructions. For detecting the concentration of IL-6/IL-8 after antibody blocking, anti-IL6/IL8 (21865-1-AP and 27095-1-AP; Proteintech) and Protein A/G Magnetic Beads (HY-K0202; MCE) were used for isolation.

### ChIP assay

The cells were crosslinked with 1% formaldehyde for 10 min at room temperature and quenched with 125 mM glycine. Sonication fragmented the crosslinked chromatin into 200–800 base pairs. Anti-STAT3 (#12640; CST), rabbit IgG (30000-0-AP; Proteintech), and Protein A/G Magnetic Beads (HY-K0202; MCE) were used to immunoprecipitate the supernatant at 4 °C overnight with corresponding antibodies. Supplementary Table S5 lists primers for amplification of purified DNA. The PCR analysis was performed as previously described [30].

### Orthotopic tongue xenograft model

Male BALB/c nude mice aged 4–5 weeks (GemPharmatech, Nanjing, China) were randomly assigned to two groups (by a single sequence of the random assignments) for the generation of an orthotopic xenograft tongue tumor model (effect size d = 1.46, α = 0.05, power = 85%, *n* = 10), experiments were carried out following animal protocols approved by the Laboratory Animal Welfare and Ethics Committee of Fujian medical university. A mixture of 10^6^ LN4 OSCC cells and 10^6^ conditional fibroblasts were suspended in 20 μL of recommended medium and injected into the anterior portion of the tongue of nude mice for 45 days (No blinding was done). From day 7 after injection, mice were given additional liquid milk powder for nutritional support. Fibroblasts with knockdown of MMP1 using lentivirus-shRNA were used for the orthotopic xenograft tongue tumor model to confirm the role of MMP1 derived from fibroblasts in tumor invasion and metastasis.

### Statistics

The statistical tests are indicated in the figure and figure legends. Data are expressed as the mean ± SD. GraphPad Prism 9, G.Power and R studio were used for Statistical analysis. Experiments were repeated independently three times, respectively, with similar results to demonstrate reproducibility.

### Supplementary information

Supplementary Materials (Table S1-S5, Figure S1-S3)

Supplementary Materials for original WB images

## Data Availability

All data in this study are included in the article and supplementary material files.
