# Supplementary material for: IL-8 activates fibroblasts to promote the invasion of HNSCC cells via STAT3-MMP1
Source: Cell Death Discov. 2024 Feb 6;10:65. doi: 10.1038/s41420-024-01833-7 (PMC10847094; doi:10.1038/s41420-024-01833-7)
Supplement: Supplementary file 1 — Supplementary Materials (Table S1-S5, Figure S1-S3) [file 41420_2024_1833_MOESM1_ESM.pdf]

Supplementary Materials for

IL-8 activates fibroblasts to promote the invasion of HNSCC cells via STAT3-MMP1

Yu Chen,#[1,2] Li Huang,#[3] Rui-Huan Gan,[1,2] Ting Lan,[1,2] Shuo Yuan,[2] Dali Zheng,*[1,2] and You-Guang Lu*[1,2]

1 Department of Preventive Dentistry, Affiliated Stomatological Hospital, Fujian Medical University, 246 Yang Qiao Middle Road, Fuzhou, 350000, China.

2 Key laboratory of Stomatology of Fujian Province, School and Hospital of Stomatology, Fujian Medical University, 88 Jiaotong Rd, Fuzhou, 350004, China.

3 Department of Oral and Maxillofacial Surgery, Affiliated First Hospital of Fujian Medical University, 20 Cha Zhong Road, Fuzhou, 350005, China.

*Corresponding author. #Contributed equally.

This file includes:

Tables S1 to S5.
Figures S1-3.
Original full length western blots

Table S1 Differential Expression Analysis in HNSCC

Gene Symbol	Gene ID	Median (Tumor)	Median (Normal)	Log2(Fold Change)	adjp
MMP1	ENSG00000196611.4	183.291	1.394	6.266	1.19E-30
MMP11	ENSG00000099953.9	40.74	0.88	4.473	2.56E-31
CA9	ENSG00000107159.12	24.92	0.255	4.368	1.18E-29
PTHLH	ENSG00000087494.15	137.053	6.891	4.129	7.12E-25
MMP9	ENSG00000100985.7	56.261	2.529	4.02	7.10E-36
COL1A1	ENSG00000108821.13	508.357	30.516	4.015	1.50E-27
LAMC2	ENSG00000058085.14	244.179	15.448	3.898	9.94E-34
ISG15	ENSG00000187608.8	333.975	21.942	3.868	9.55E-31
MMP3	ENSG00000149968.11	28.779	1.064	3.851	2.18E-15
SPP1	ENSG00000118785.13	65.489	3.974	3.741	5.41E-15

Table S2 The expression of MMP1 in paired tongue cancer samples

Characteristics	Cases	-	+	++	Z value	P value
Cancer	92	13	56	23		
Normal	92	60	32	0	-7.006	<0.001

Table S3 Primers used for real-time quantitative PCR analysis.

Gene	Foward primer(5'-3')	Reverse primer(5'-3')
MMP1	GGGGAGATCATCGGGACAACTC	AGAATGGCCGAGTTCATGAGCT
GAPDH	CATCCCAGAGCTGAACG	CTGGTCCTCAGTGTAGCC

Table S4 Sequences used for RNAi transfection

Gene	sense(5'-3')	antisense(5'-3')
MMP1-972	GCGUGUGACAGUAAGCUAATT	UUAGCUUACUGUCACACGCTT
MMP1-1071	CCGGAAGUUGAGCUCAAUUTT	AAUUGAGCUCAACUUCCGGTT
siRNA-NC	UUCUCCGAACGUGUCACGUTT	ACGUGACACGUUCGGAGAATT

Table S5 Primers used for ChIP assay.

Gene	Foward primer	Reverse primer
IRF1-promoter	CACGTCTTGCCTCGACTAAGG	AGTGTTTGGATTGCTCGGTG
Chr11-none	CTTGACCTTGACCTCACCCC	TGGCATTTGAACCCACGTCT
MMP1-promoter	CCCTTCCAGAAAGCCAGAGG	GAGTGTGTCTCCTTCGCACA

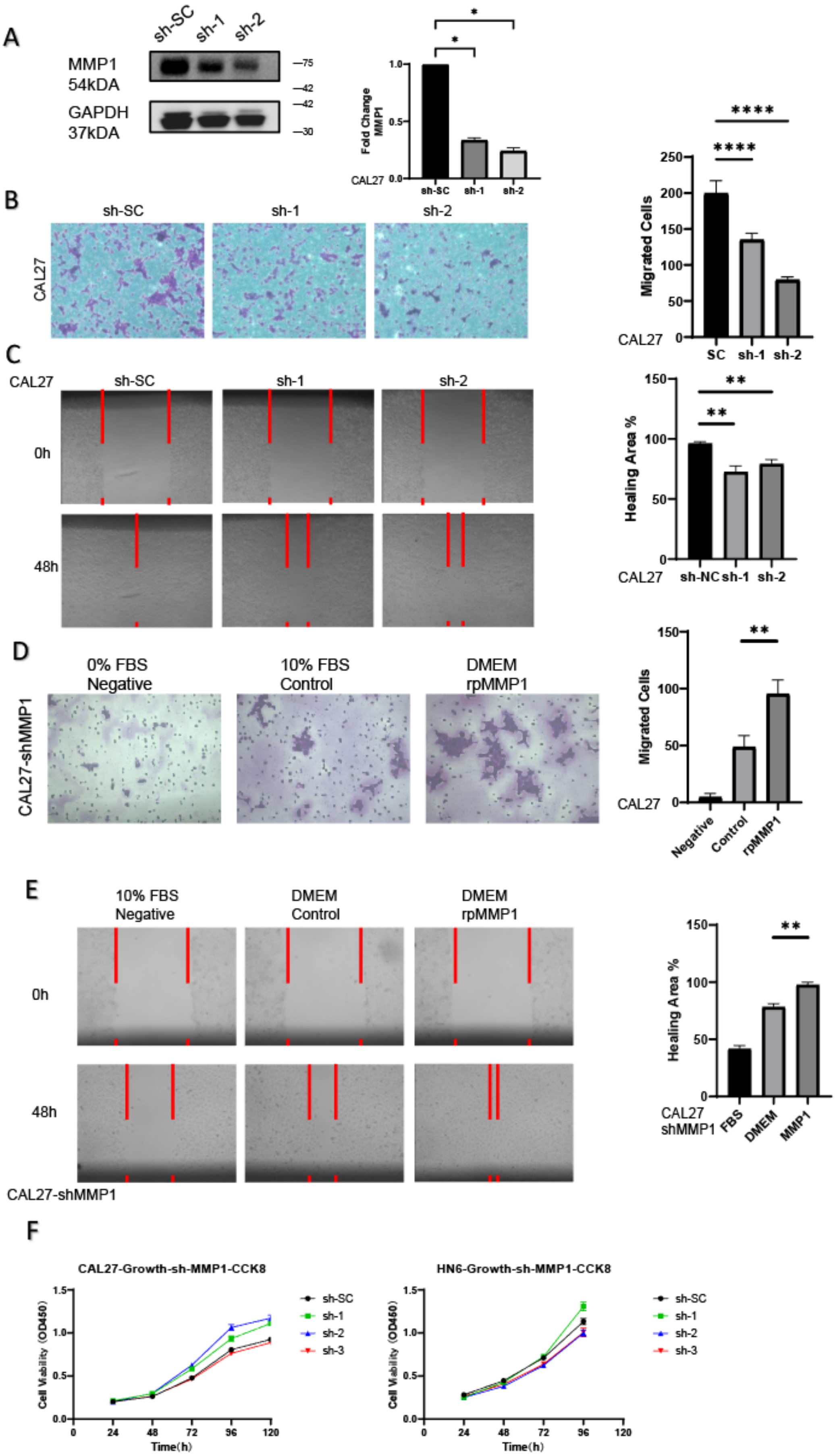
A
sh-SC
sh-1
sh-2
MMP1
54kDA
GAPDH
37kDA
75
42
42
30
Fold Change MMP1
CAL27
B
sh-SC
sh-1
sh-2
CAL27
Migrated Cells
SC
C
CAL27
0h
48h
Healing Area %
sh-NC
D
0% FBS
Negative
10% FBS
Control
DMEM
rpMMP1
CAL27-shMMP1
Negative
Control
rpMMP1
E
10% FBS
Negative
DMEM
Control
DMEM
rpMMP1
0h
48h
CAL27-shMMP1
CAL27
shMMP1
FBS
DMEM
MMP1
F
CAL27-Growth-sh-MMP1-CCK8
HN6-Growth-sh-MMP1-CCK8
Cell Viability (OD450)
Time(h)
sh-SC
sh-1
sh-2
sh-3

Fig.S1 Knockdown of MMP1 inhibits HNSCC cells migration in vitro

(A,) The transcriptional and translational levels of MMP1 were detected by qRT-PCR and western blotting in HNSCC cell line (CAL27); the migration abilities of HNSCC cells with MMP1 knockdown were detected using Transwell assays (B) and wound-healing assays (C) respectively; after MMP1 knockdown, the migration abilities of HNSCC cells treated with rhMMP1 at 48h, 20 ng/ml were detected using Transwell assays (D) and wound-healing assays (E) respectively; (F) the proliferation ability of HNSCC cells was measured by the CCK8 assay.

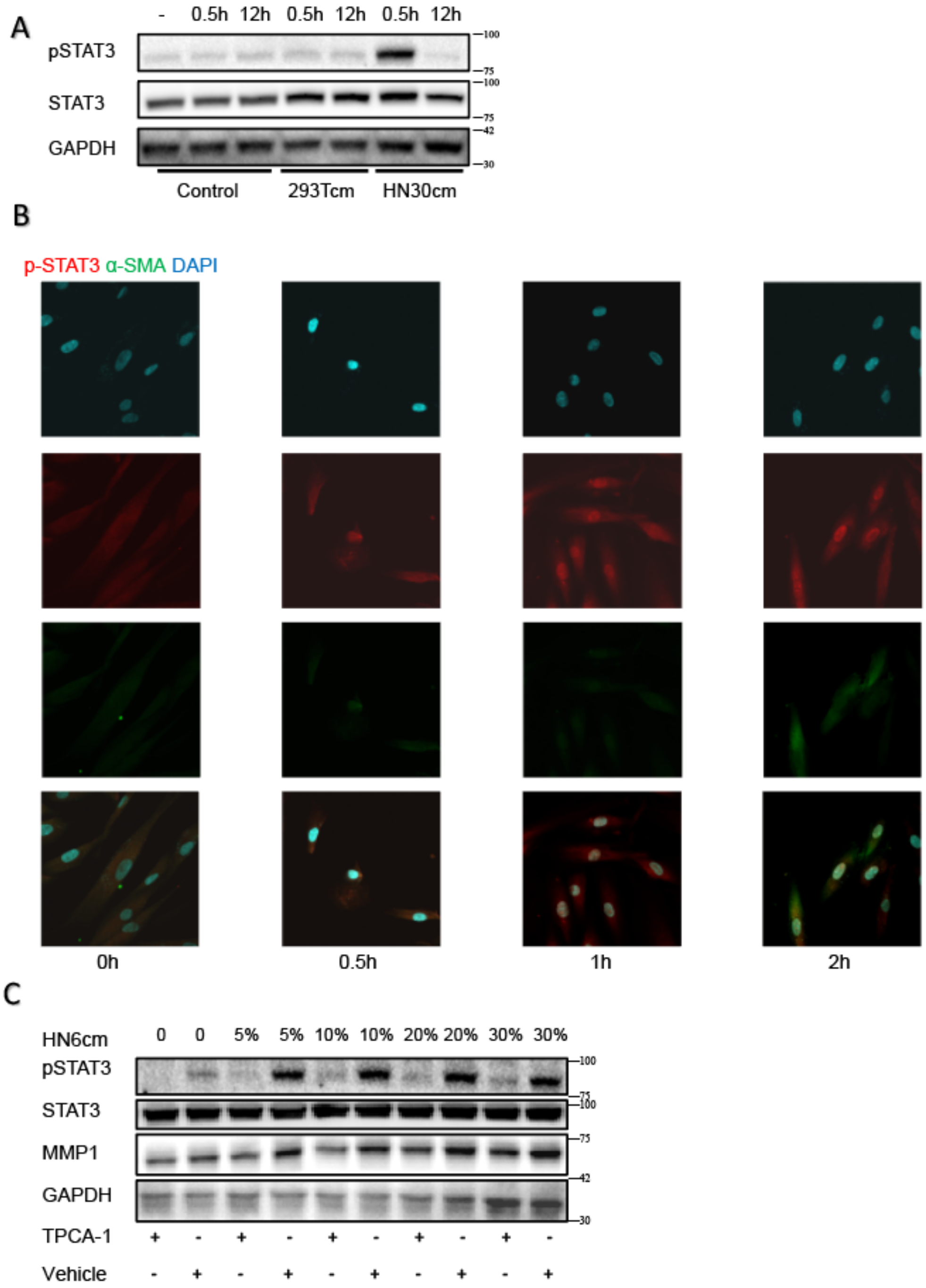

Fig.S2 STAT3 signaling was activated by the conditioned medium of HNSCC cells (A) HNSCC cell lines upregulated the expression of MMP1 in NFs while the level of phospho-STAT3 protein increased in 0.5 h; then (B) phospho-STAT3 protein located in nuclear was detected using confocal laser scanning microscope. (C) HN6-CM increased the expression of MMP1 protein in dose-dependent manner in 12 hours, which was then suppressed by TPCA-1.

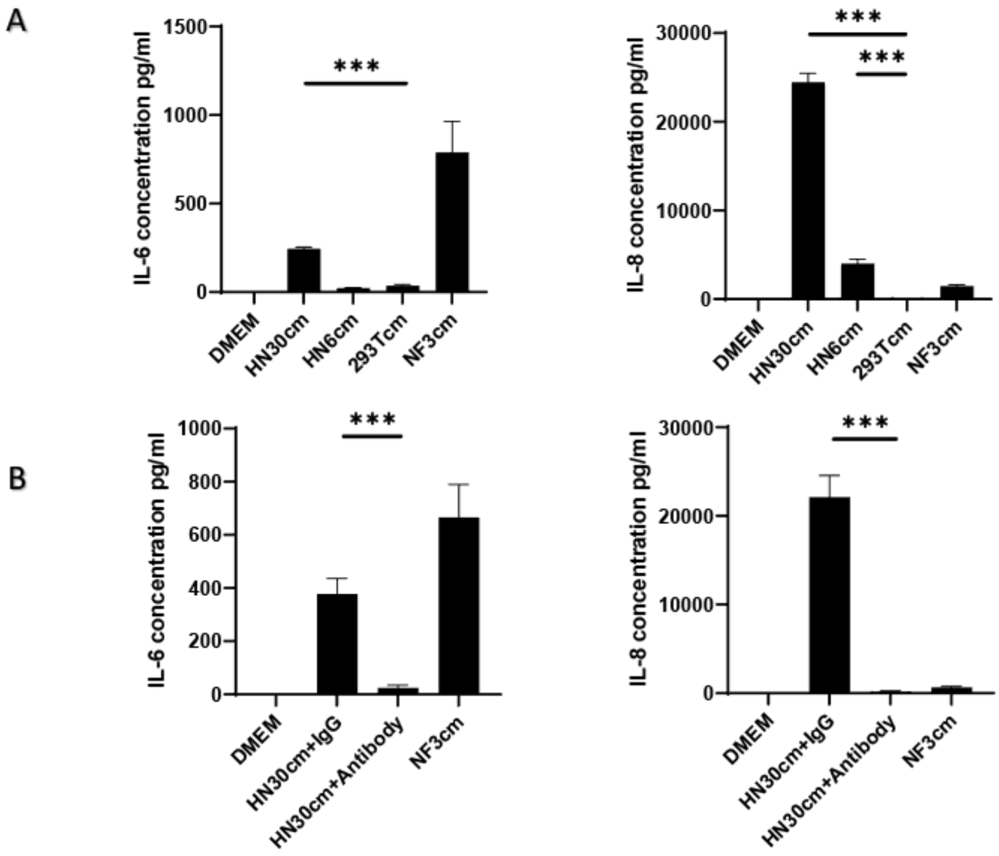

Fig.S3 ELISA assays for conditioned medium.

The levels of IL-6 and IL-8 were determined by ELISA. (A) after cells were cultured with DMEM and starved for 24 h. (B) HN30 conditioned medium was collected, IgG, IL-6 antibody (100 ng/ml) or IL-8 antibody (100 ng/ml) was used for binding antigens, followed by protein A/G magnetic beads for isolating antibodies from conditioned medium.
