# Supplementary material for: IL-8 activates fibroblasts to promote the invasion of HNSCC cells via STAT3-MMP1
Source: Cell Death Discov. 2024 Feb 6;10:65. doi: 10.1038/s41420-024-01833-7 (PMC10847094; doi:10.1038/s41420-024-01833-7)
Supplement: Supplementary file 2 — Supplementary Materials for original WB images [file 41420_2024_1833_MOESM2_ESM.pdf]

Supplementary Materials for

IL-8 activates fibroblasts to promote the invasion of HNSCC cells via STAT3-MMP1

This file includes:
Original full length western blots

Original full length western blots

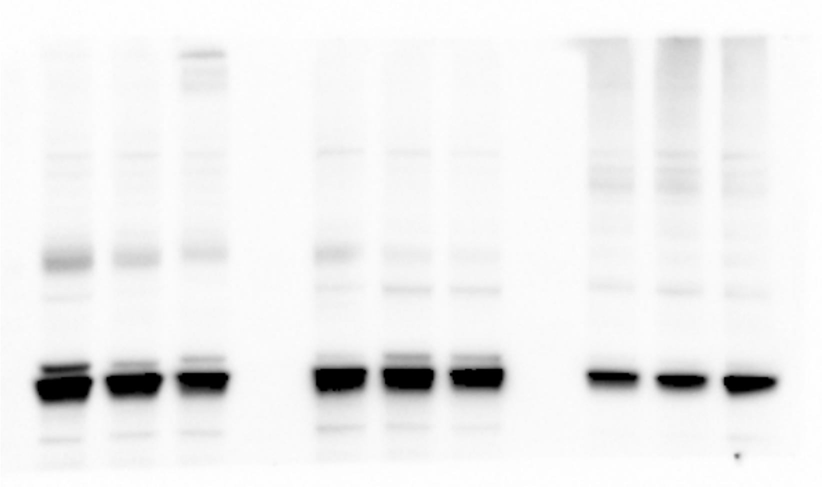

Fig2B-GAPDH

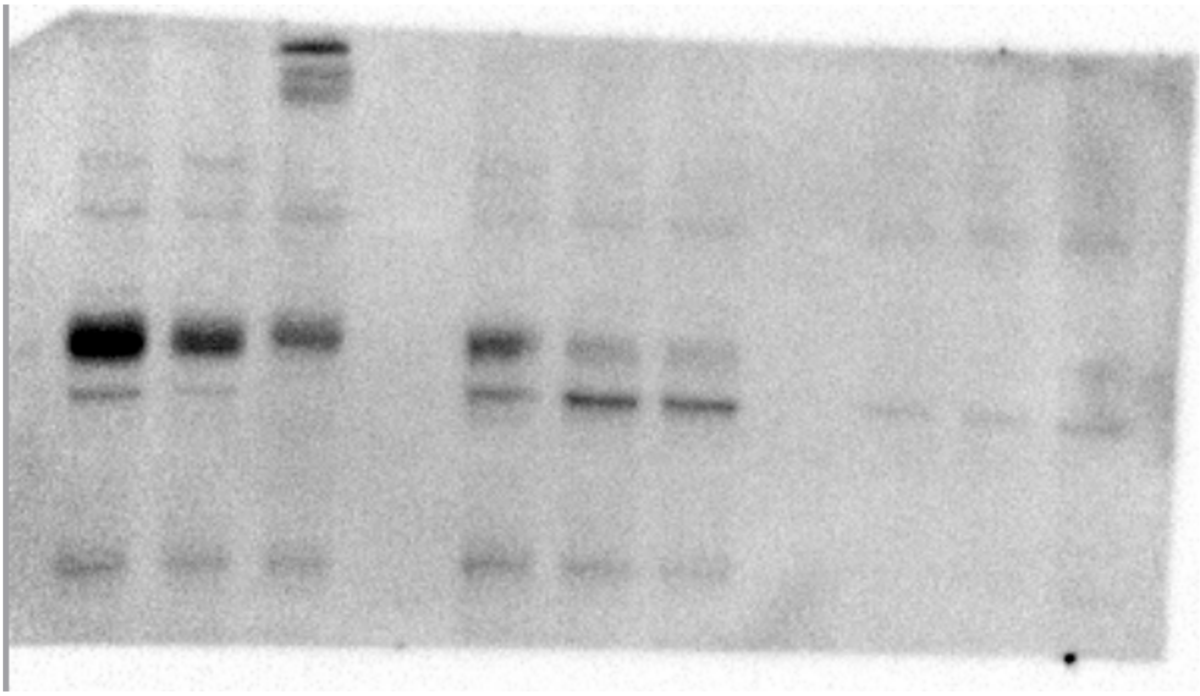

Fig2B-MMP1

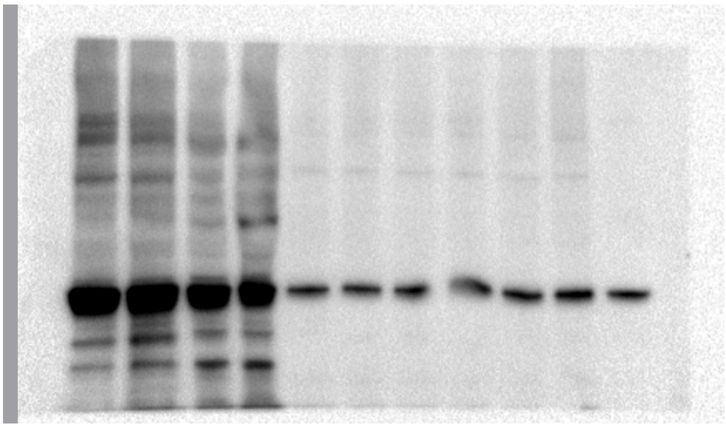

Fig3C-GAPDH

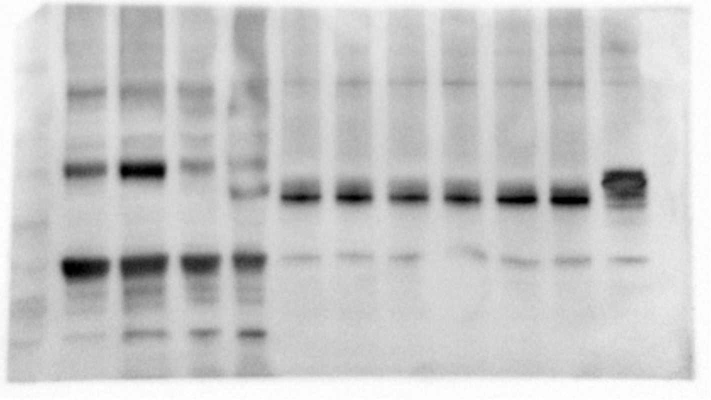

Fig3C-MMP1

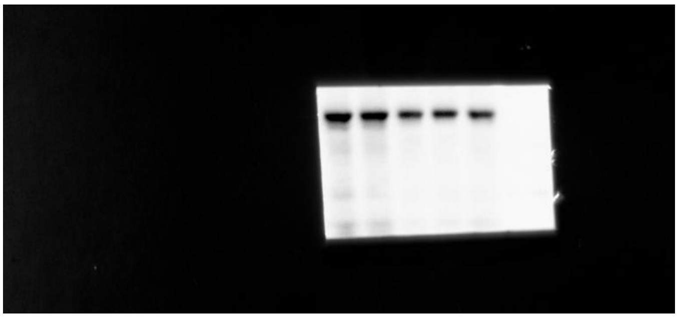

Fig3E-GAPDH

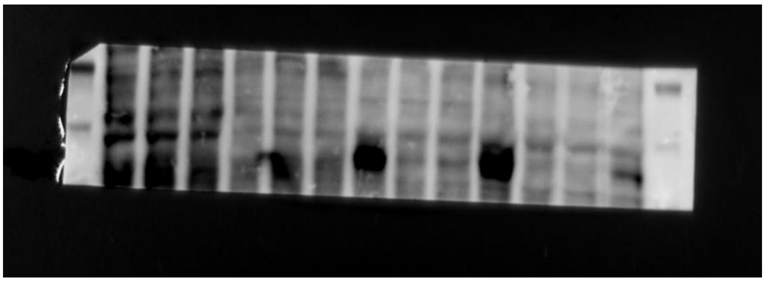

Fig3E-MMP1

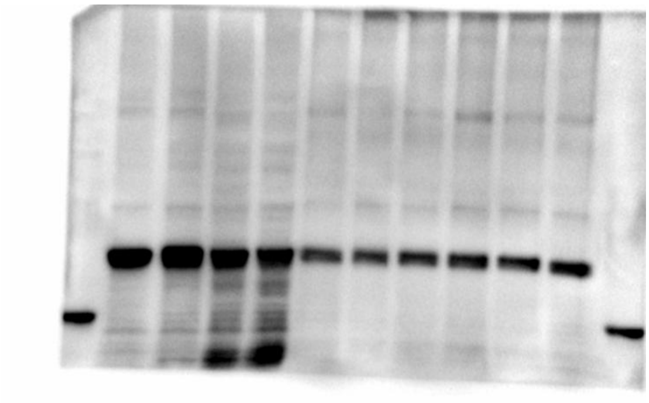

Fig4A-GAPDH

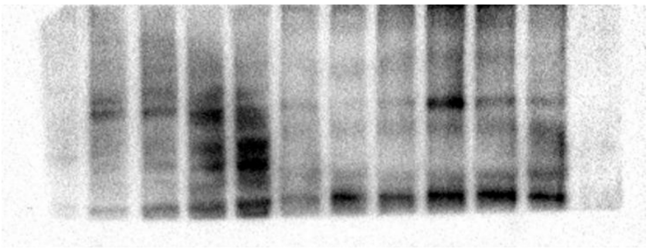

Fig4A-MMP1

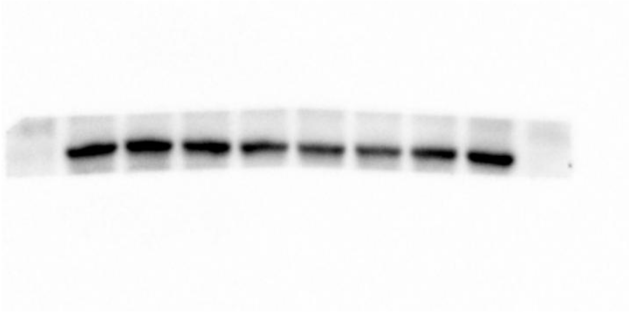

Fig4B-GAPDH

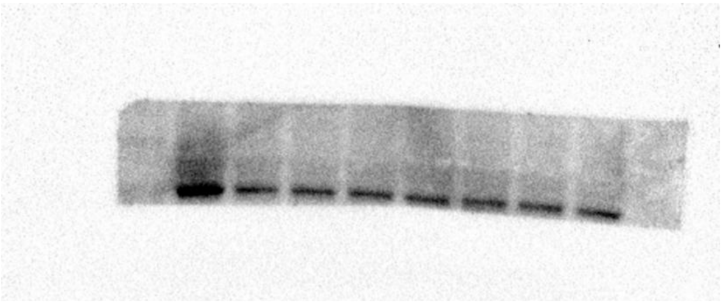

Fig4B-MMP1

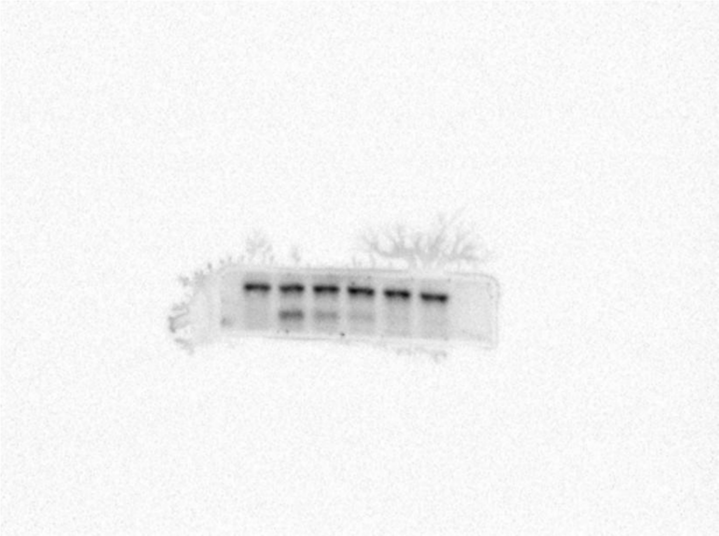

Fig4E-GAPDH

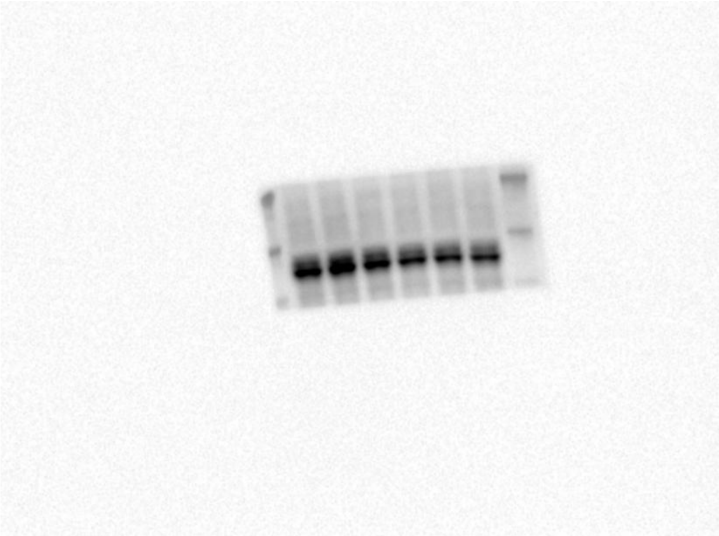

Fig4E-MMP1

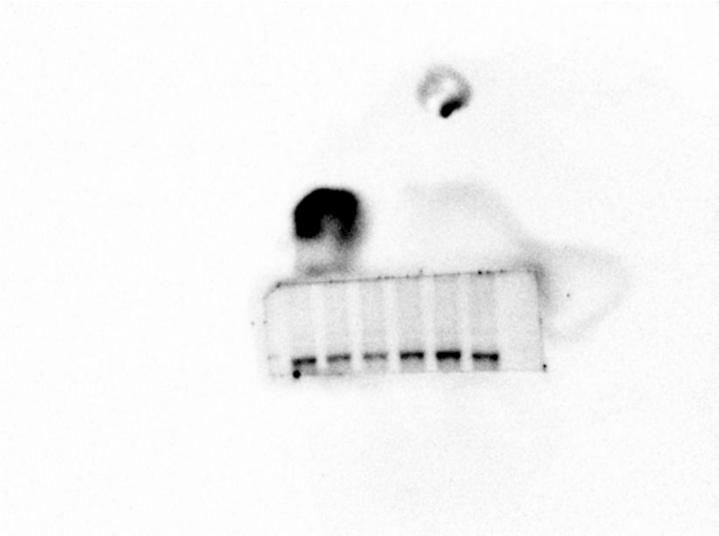

Fig4E-PSTAT3

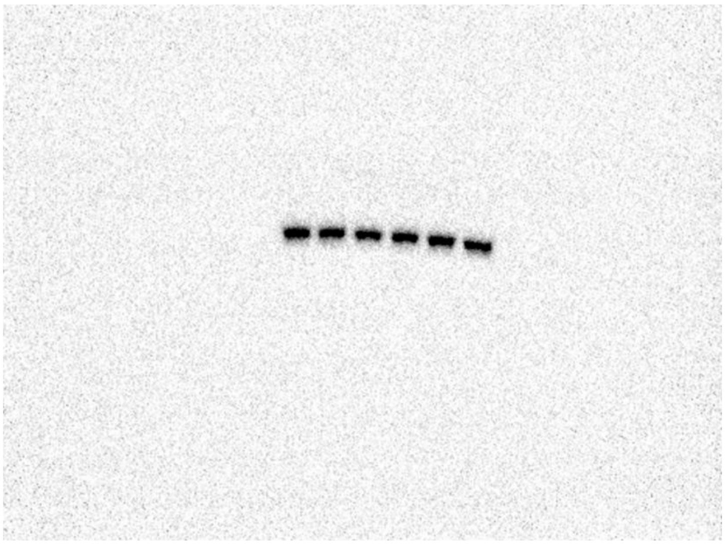

Fig4E-STAT3

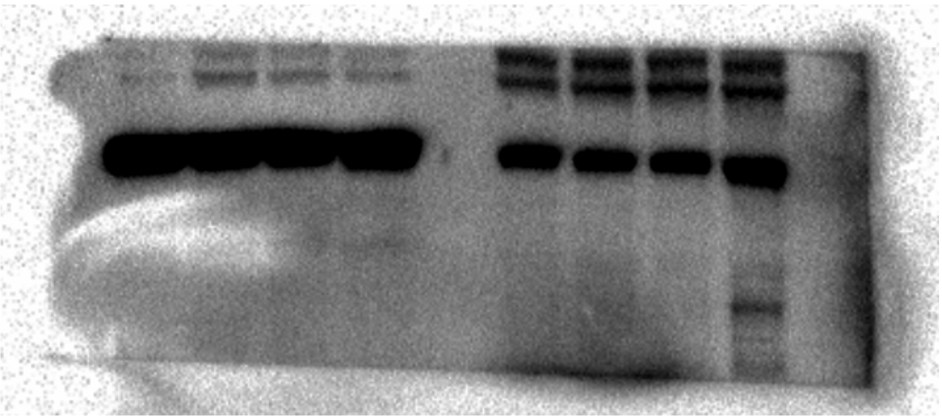

Fig4F-LaminAC-Tubulin

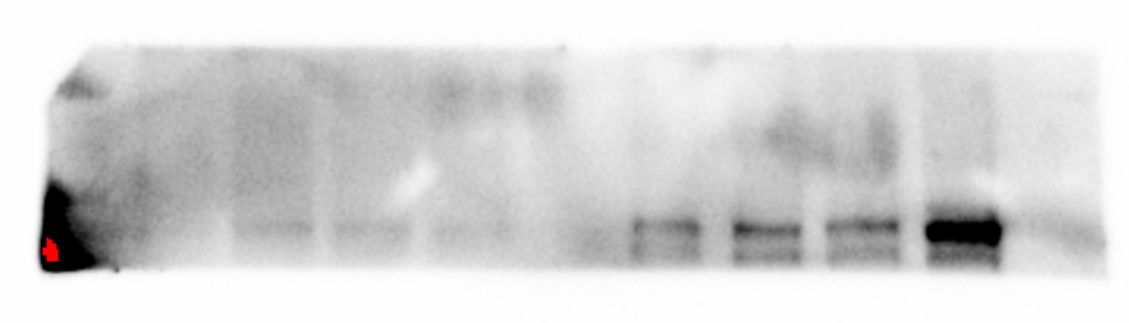

Fig4F-pSTAT3

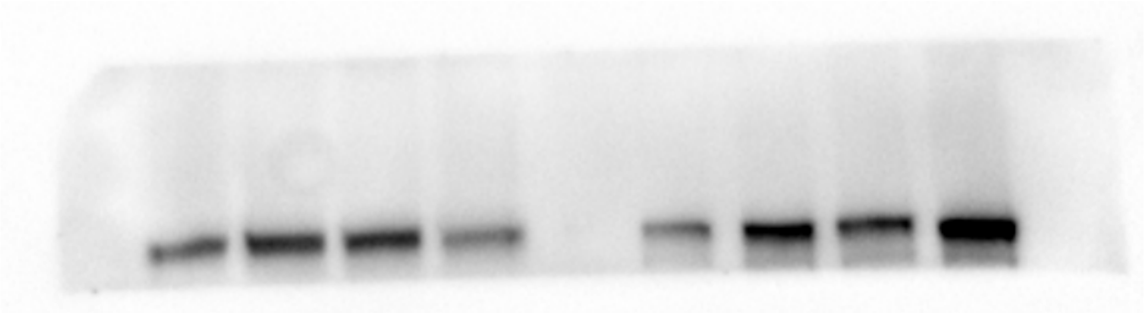

Fig4F-STAT3

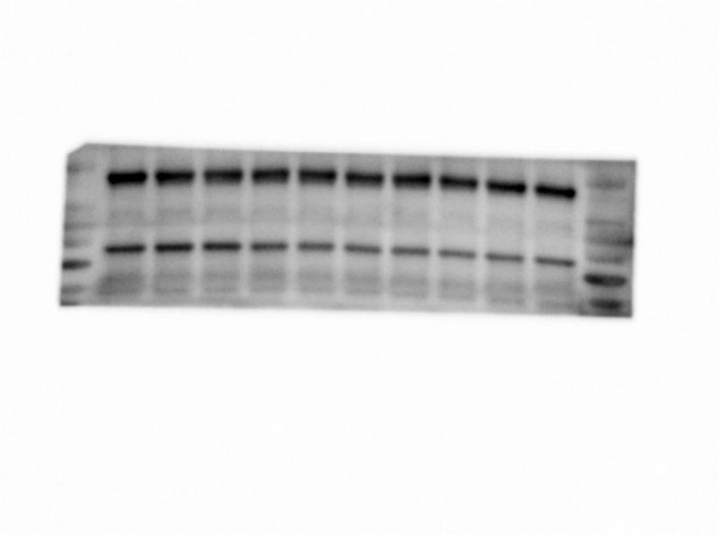

Fig5A-GAPDH

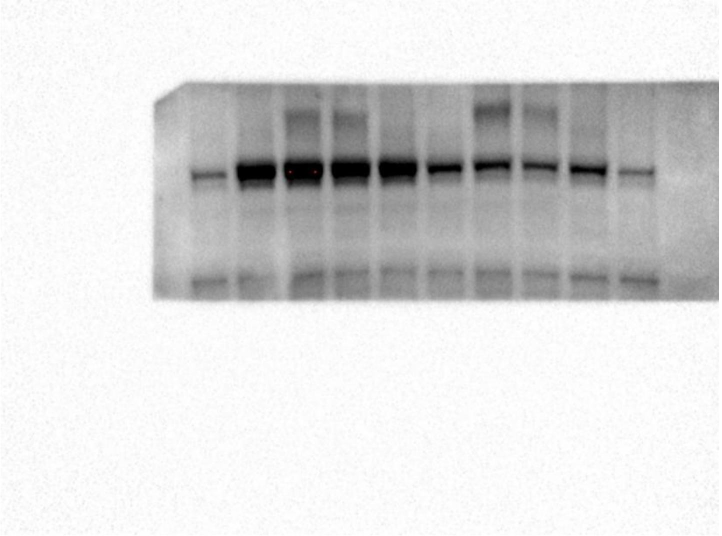

Fig5A-PSTAT3

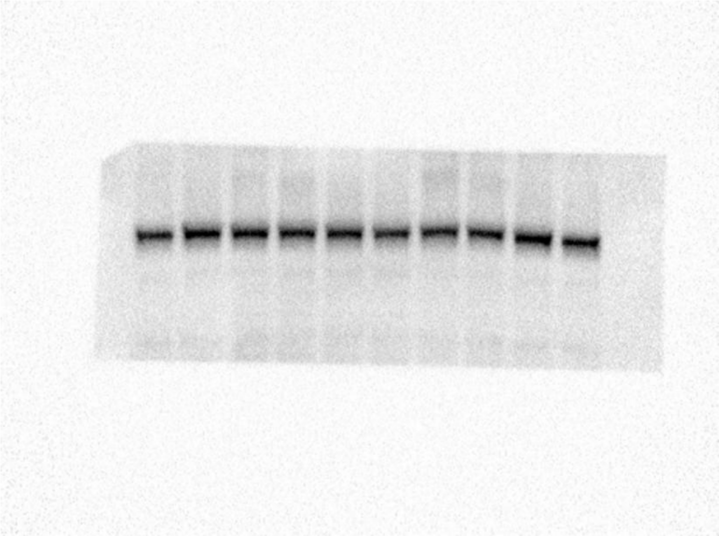

Fig5A-STAT3

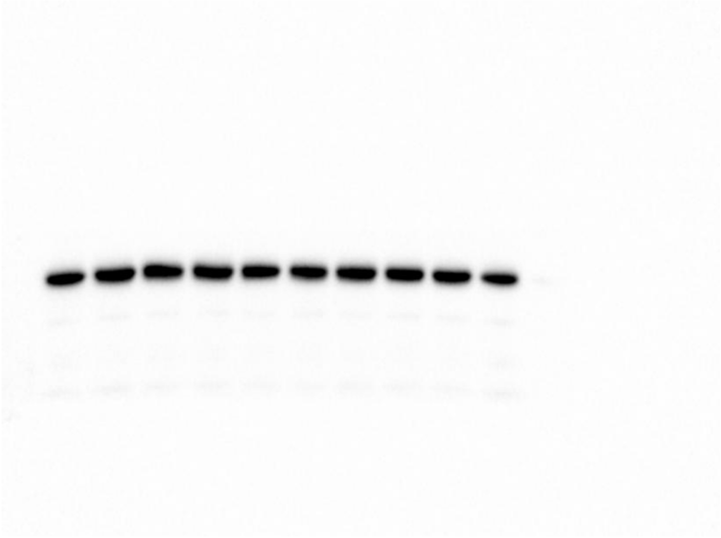

Fig5B-GAPDH

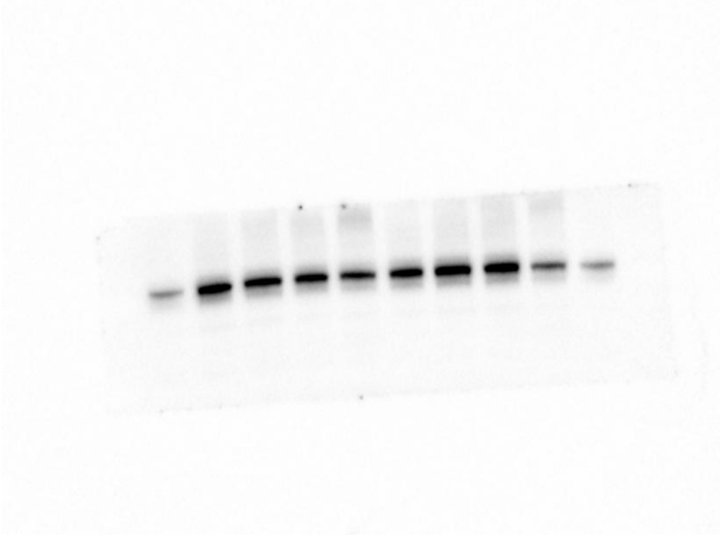

Fig5B-PSTAT3

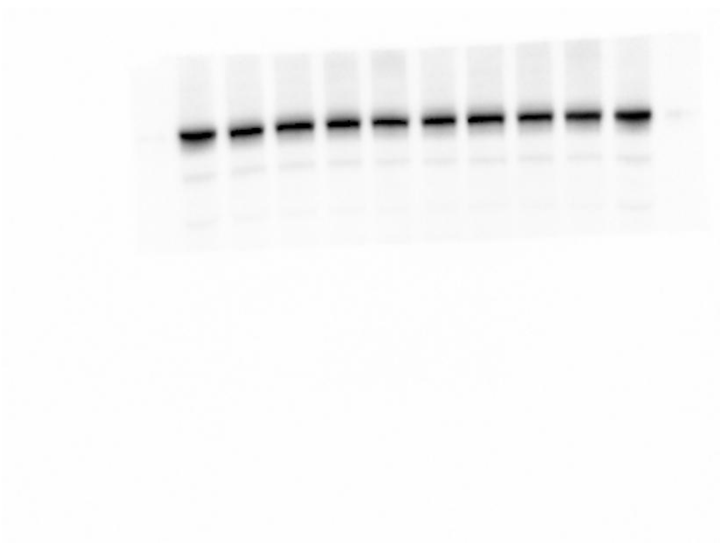

Fig5B-STAT3

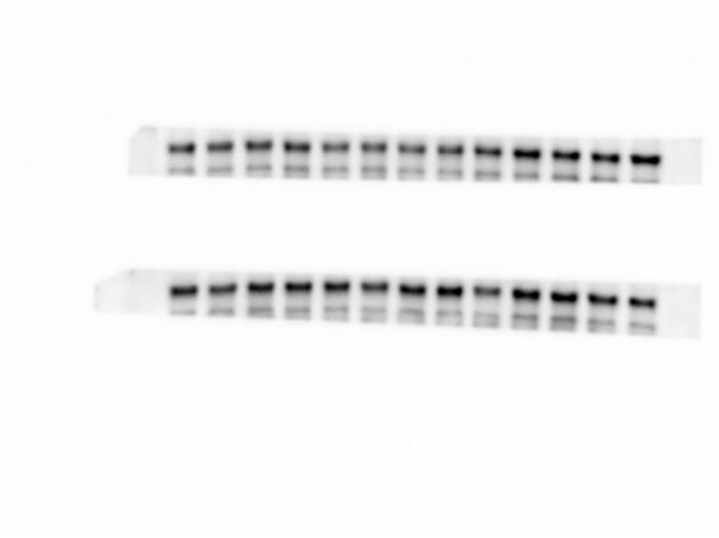

Fig5C-GAPDH

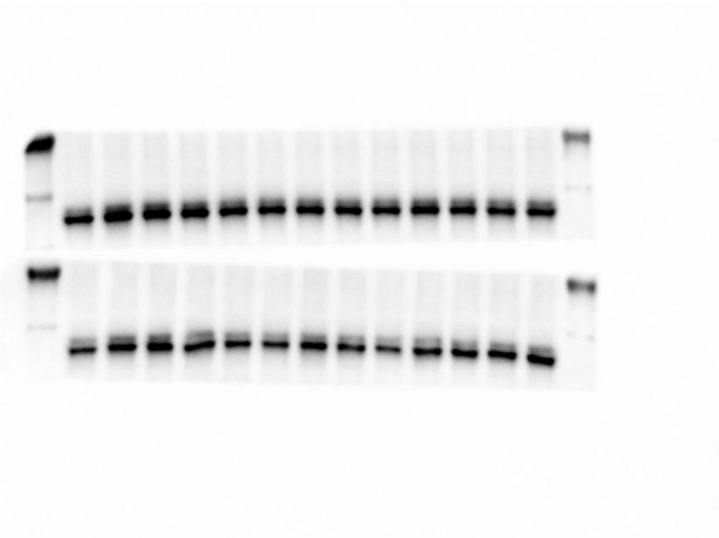

Fig5C-MMP1

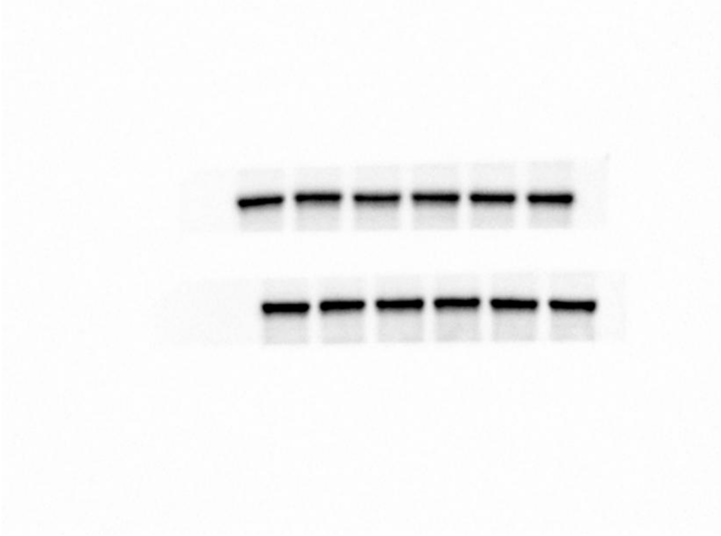

Fig6A-G3P-N6-30

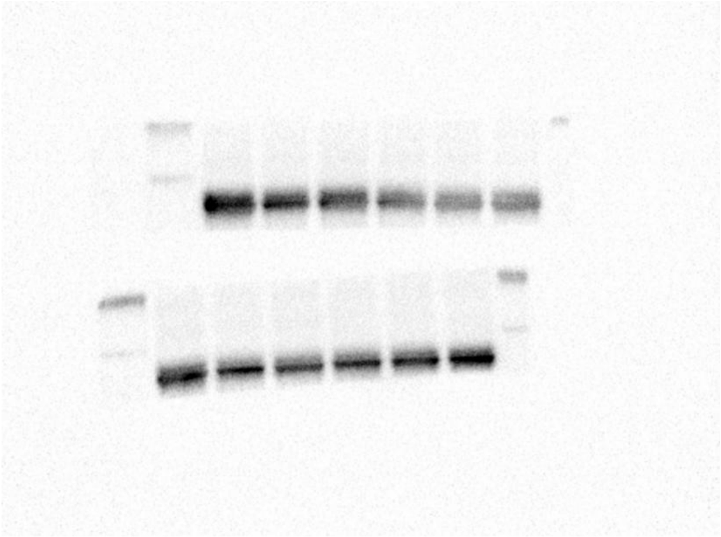

Fig6A-MMP1-30-N6

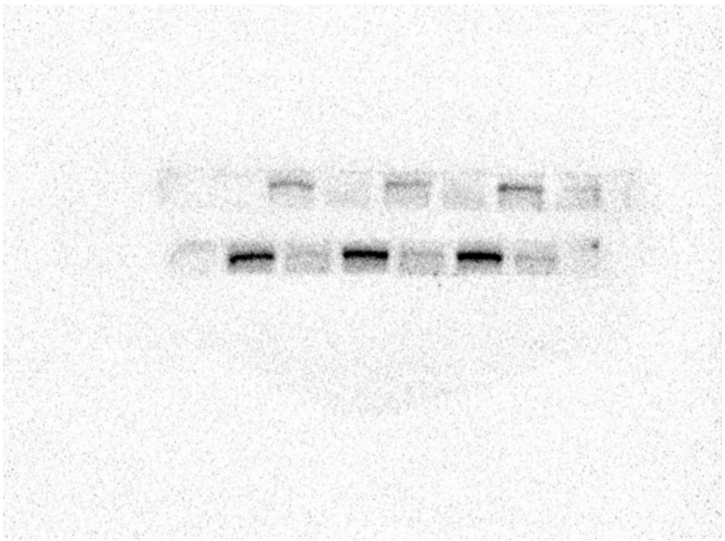

Fig6A-P-STAT3

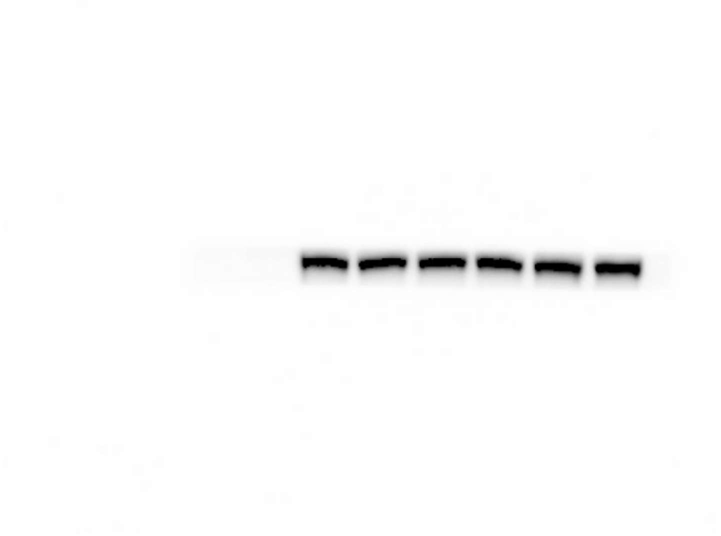

Fig6A-STAT3-30

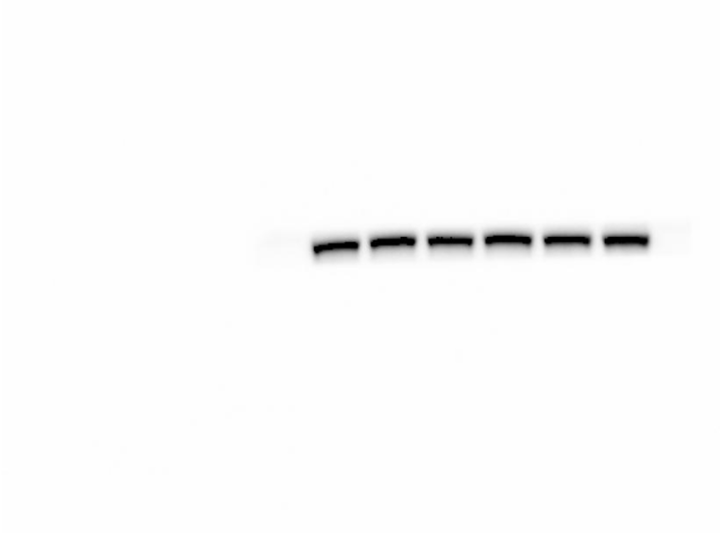

Fig6A-STAT3-N6

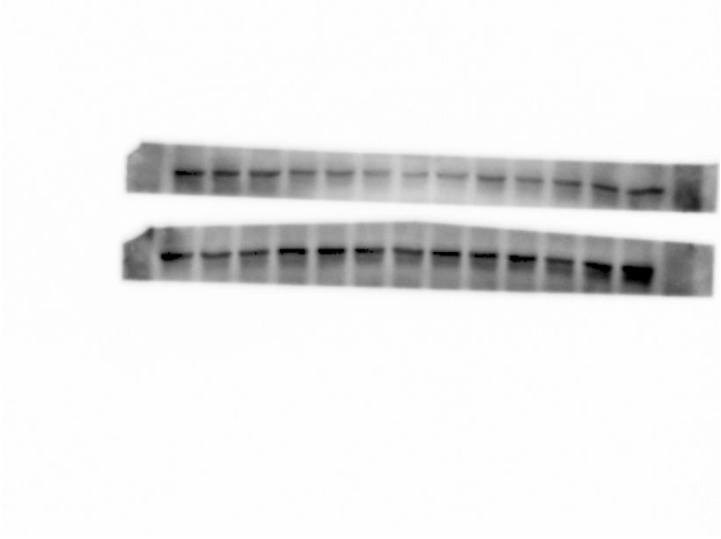

Fig6B-G3P-N6-30

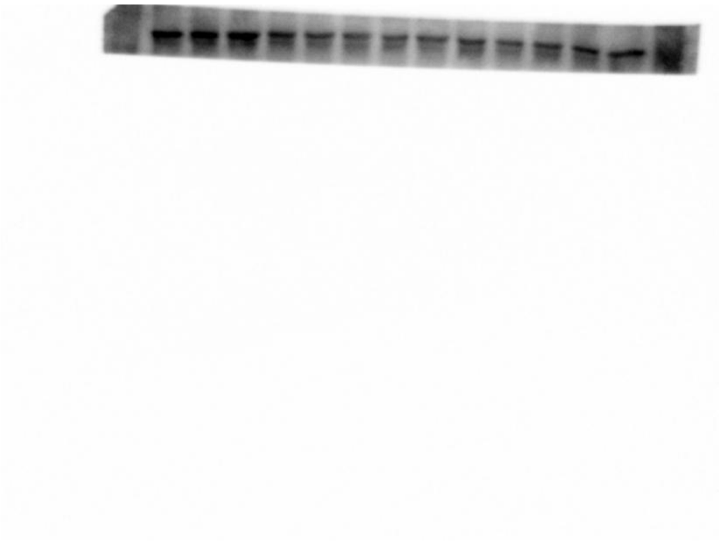
Fig6B-GAPDH
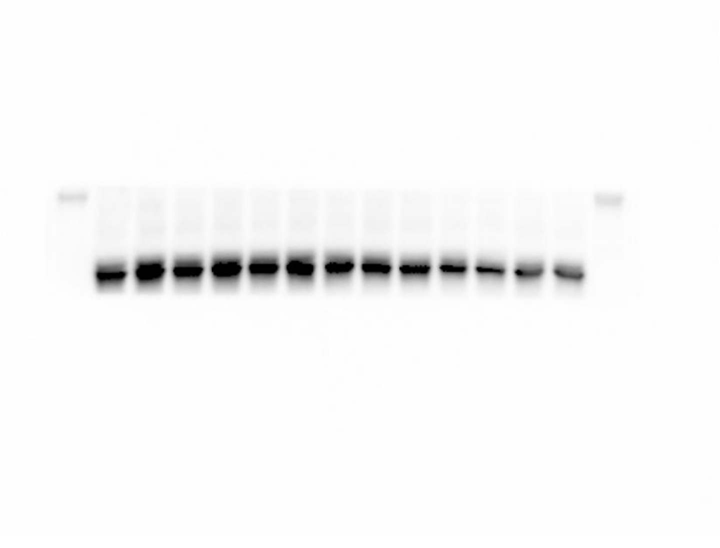
Fig6B-MMP1
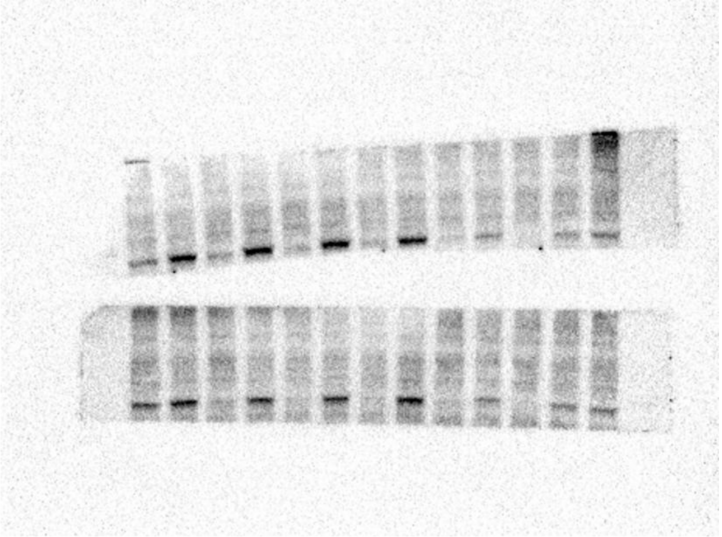
Fig6B-pSTAT3

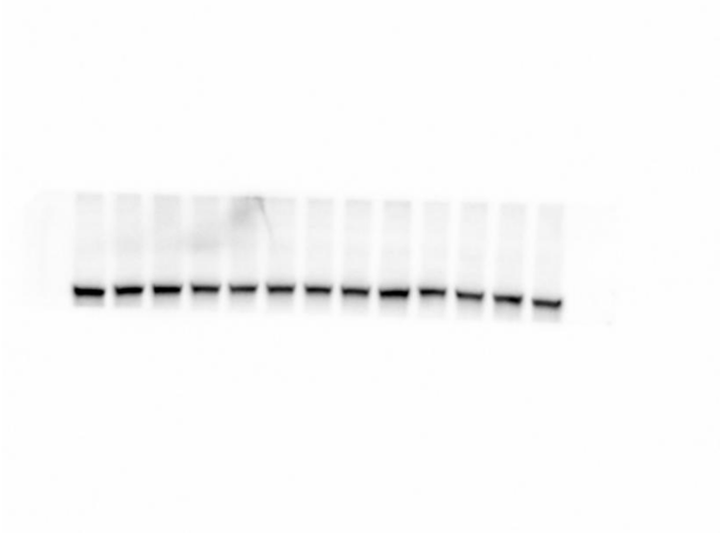

Fig6B-STAT3

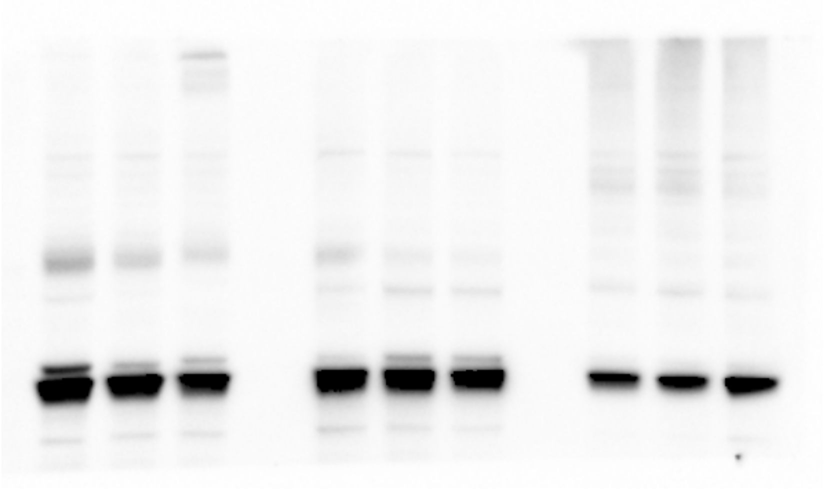

FigS1A-GAPDH

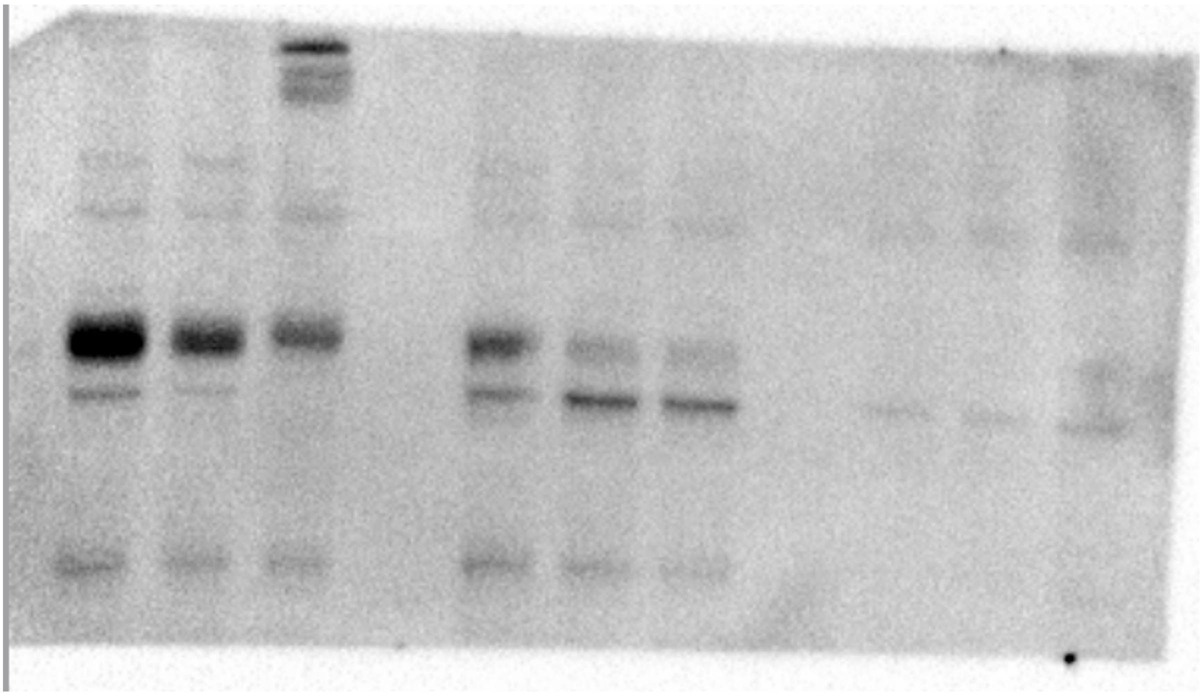

FigS1A-MMP1

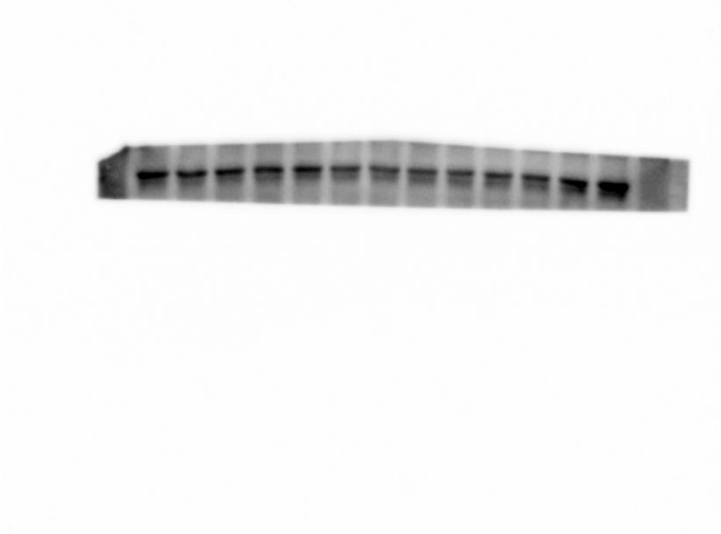

FigS2-GAPDH

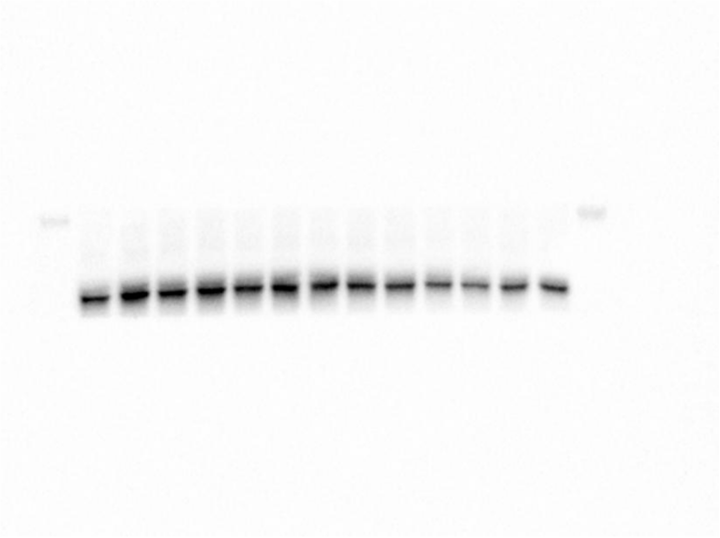

FigS2-MMP1

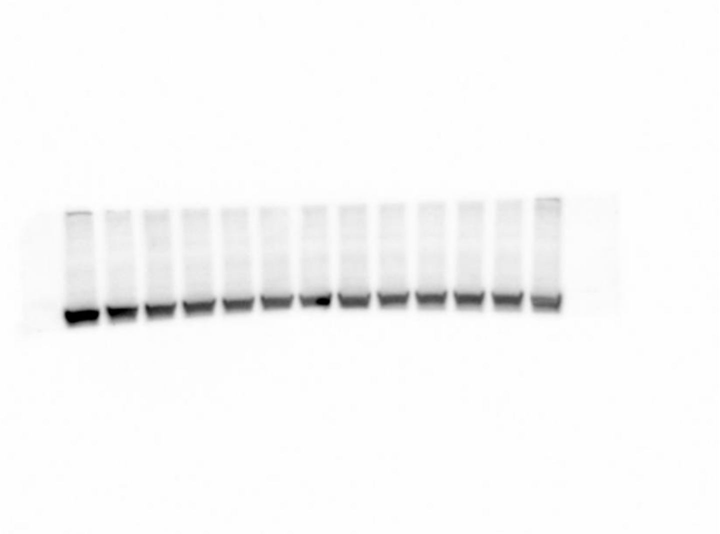

FigS2-STAT3
